# Towards safer colorectal surgery worldwide: Outcomes and benchmarks from the ESCP CORREA 2022 audit

**DOI:** 10.1111/codi.70281

**Published:** 2025-11-24

**Authors:** 2022 European Society of Coloproctology (ESCP) CORREA Collaborating Group, Ionut Negoi, Gabrielle van Ramhorst, Shaji Sebastian, Gianluca Pellino, Muhammed Elhadi, Audrius Dulskas, Bryar Kadir, James Glasbey, Peter Neary, Ana María Minaya Bravo, James Keatley, Alaa El‐Hussuna, Thomas Pinkney, Sanjay Chaudhry, Laura Magill, Rita Perry, Sue Blackwell, Sanjay Chaudhri, Sharfuddin Chowdhury, Dragomir Dardanov, Caterina Foppa, Gaetano Gallo, Elizabeth Li, Dion Morton, Francesco Pata, Gabrielle van Ramshorst, Beatriz Silva Mendes, Baljit Singh, Erman Aytac, Stephanie Breukink, Pamela Buchwald, Niki Christou, Nir Horesh, Karoline Horisberger, Jim Khan, Laura Koskenvuo, Yurij Košir, Hans Lederhuber, Mostafa Shalaby, Carolynne Vaizey, S. Bilali, S. Ferko, A. Gjata, E. Shehi, J. Shahu, S. Bouaoud, M. Abdoun, K. Bouchenak, H. Saada, A. Ouyahia, M. Rais, E. Seddik, M. Bouaoud, A. Kouicem, N. Yacine, L. Nawel, N. Talbi, A. Tidjane, B. Tabeti, A. Hebbar, I. Lalmi, A. Bouanani, O. Bali, N. Benallel, C. Chwat, G. Lemme, G. Rosato, F. Alexandre, D. Valli, M. Gosselink, D. Daryanani, J. Smit, A. Ponson, Y. Casseres, N. Strugnell, B. D’Souza, S. Badiani, T. Kuany, F. Aigner, M. Mitteregger, S. Uranitsch, C. Allmer, G. Moitzi, E. Wallner, R. Stadler, F. Herbst, I. Iliev, E. Samadov, A. Ibrahimli, M. Saeed, R. Varkey, B. Ebrahim, H. Aljazaf, I. Juma, B. Monami, S. Markiewicz, D. Francart, F. Jehaes, N. Debergh, P. Malvaux, S. Grandjean, J. Stijns, E. Van Eetvelde, T. Van de Winkel, S. Violon, G. van Ramshorst, S. Tavares Sousa Castro, D. Van de Putte, Y. Van Nieuwenhove, P. Pattyn, T. Ivanov, E. Filipov, D. Georgiev, V. Neykov, D. Dardanov, M. Sokolov, S. Maslyankov, S. Toshev, P. Gribnev, A. Koychev, M. Karamanliev, D. Dimitrov, P. Vladova, M. Shoshkova, A. Shanker, M. Slavchev, N. Belev, R. Penkov, B. Atanasov, P. Krastev, I. Yotsov, G. Šantak, N. Gouvas, P. Papatheodorou, K. Konstantinou, G. Kokkinos, A. Stylianou, P. Christensen, R. Balachandran, J. Eriksen, M. Kristensen, S. Ingvarsdóttir, M. Ellebaek, J. Baelum, M. Kjær, P. Cuk, I. Al‐Najami, U. Løve, P. Fink, T. Eliasen, D. Zavrakidis, S. Lynge, L. Sjælland, M. Abdel‐Maboud, S. Mahmoud, H. Abozied, M. Al Sayed, A. Swealem, A. Elshinnawy, A. Amer, M. Ewedah, M. Shalaby, H. Elfeki, A. Sakr, A. Sanad, M. Sadek, K. Abdelwahab, I. Nazif, H. Aboelfarh, B. Refky, M. Shetiwy, I. Metwally, M. Abdelkhalek, M. Zuhdy, M. Omar, A. Safy, M. Alansary, B. Trilling, J. Faucheron, F. Tidadini, L. Audeguy, Q. Chenevas‐Paule, A. Roudier, N. de Angelis, C. Schena, G. Bianchi, N. Christou, A. Fürst, M. Brunner, S. Blümel, V. Schmidt, A. Krappitz, A. Türler, M. Derenbach, W. Lydia, J. Baral, S. Muench, F. Pullig, R. Langbecker, D. Jauch, S. Fichtner‐Feigl, H. Neeff, C. Berlin, L. Matuschik, J. Kleeff, U. Ronellenfitsch, O. Bayram, J. Klose, S. Rieder, D. Korkolis, A. Sarafi, E. Fradelos, G. Kavalieratos, I. Katsoulis, I. Papaconstantinou, M. Konstadoulakis, T. Theodosopoulos, A. Gklavas, D. Politis, T. Petropoulou, A. Polidorou, A. Ioannidis, M. Konstantinidis, S. Konstantinidou, K. Konstantinidis, D. Manatakis, C. Barkolias, D. Balalis, N. Tasis, S. Kapiris, A. Kolinioti, M. Sotiropoulou, M. Psarologos, P. Metaxas, A. Papadopoulos, E. Manioti, O. Mouzakis, D. Broutas, V. Nikolaou, G. Tzovaras, I. Baloyiannis, K. Perivoliotis, I. Mamaloudis, F. Bobou, K. Bouchagier, G. Skroubis, I. Maroulis, F. Mulita, E. Abalov, O. Ioannidis, L. Loutzidou, E. Anestiadou, S. Bitsianis, S. Symeonidis, G. Theodoropoulos, M. Frountzas, S. Volteas, N. Dimitriou, D. Thermou, G. Karantzikos, S. Kympouris, C. Mavrantonis, P. Alexakou, A. Karagouni, N. Boltsis, D. Paradellis, D. Schizas, I. Karavokyros, N. Machairas, A. Syllaios, S. Kykalos, K. Stamou, G. Tsiotos, D. Fanidou, E. Kontis, I. Katsaros, E. Kaouras, L. Katsiaras, P. Manikis, N. Zampitis, A. Marinis, E. Bourmpouteli, M. Merrakos, F. Stefou, B. Banky, P. Ónody, L. Lakatos, Á. Dániel, V. Bencze, A. Fülöp, E. Valsdottir, J. Atladottir, H. Sigurdsson, H. Einarsdottir, P. Moller, A. Saklani, M. Kazi, D. Kearney, D. Petrocz‐Turu, C. O’Reily, S. Martin, R. Kennelly, F. Lecot, I. Reynolds, D. Winter, A. Hanly, S. Arya, D. Hechtl, J. O’Riordan, É. Ryan, J. Mahon, D. Kavanagh, N. Christodoulou, P. Neary, B. Creavin, F. Cooke, P. McCullough, H. Earley, I. Kent, N. Horesh, A. Mansur, M. Kyzer, R. Antebi, R. Tutino, M. Santarelli, D. Visconti, A. Ferguglia, L. Rorato, M. Annecchiarico, G. Argenio, C. Pedrazzani, G. Turri, C. Conti, G. Gecchele, E. De Giulio, N. Bicelli, R. Balestri, M. Puccini, P. Buccianti, R. D’Ischia, D. Pezzati, L. Marano, L. Carbone, L. Verre, F. Roviello, R. Piagnerelli, D. Scala, B. Puppio, P. Maione, G. Marino, B. Scotto, F. Ascari, S. Muratori, I. Roli, V. Tonini, M. Cervellera, L. Sartarelli, A. Lauretta, S. Pollesel, M. Olivieri, C. Belluco, M. Calabrò, A. Muratore, B. Cuzzola, E. Herranz Van Nood, M. De Zuanni, M. Manigrasso, G. De Palma, M. Milone, S. Vertaldi, A. Marello, L. Solaini, A. Luzzi, E. Romairone, S. Carrabetta, P. Grondona, A. Filippelli, G. Moretto, I. Harmony, F. Bianco, S. Gili, A. Novi, S. Grassia, A. Cappiello, N. Tamini, L. Cigagna, F. Ghignone, G. Ugolini, L. Santandrea, S. Bolzon, C. Marafante, E. Moggia, M. Grande, M. Campanelli, L. Siragusa, F. Santori, G. Sica, U. Grossi, R. Galleano, A. Langone, M. Ciciliot, M. Malerba, P. De Nardi, A. Vignali, G. Carbone, R. Rosati, M. Rottoli, A. Belvedere, A. Gori, C. Larotonda, S. Cardelli, T. Violante, D. Sasia, M. Giuffrida, E. Beltrami, G. Preve, S. Armentano, P. Mosangini, M. Ammendola, G. Currò, G. Ammerata, R. Filippo, A. Picciariello, M. De Fazio, L. Vincenti, G. Tomasicchio, F. Selvaggi, G. Pellino, M. D’Ambrosio, F. Menegon Tasselli, D. Massaro, G. Terrosu, G. Calini, L. Bonello, F. Leone, A. Khamees, S. Al‐issawi, S. Abu Wardeh, A. Mansour, A. Mohammad, O. Shattarah, R. Ebbini, O. Sarhan, M. Nour, F. Daoud, A. Tarboush, A. Pcolkins, G. Alkadeeki, F. AlMaadany, A. Kredan, A. Mohamed, A. Haddud, W. Abdulnabi, F. Fauzi, M. Ali, D. Zreeg, W. Aldressi, E. Hosain, H. Alkeelani, I. Abuzeid, A. Alkaseek, H. Shames, H. Baliad Bakeer, F. Elhajdawe, E. Abdulwahed, E. Alshareea, K. Derwish, E. Soula, M. Zagmen, S. Zagmen, A. Fteis, H. Mansour, H. Gholka, A. Dulskas, J. Kuliavas, Z. Gricius, A. Aleinikov, R. Bradunaite, A. Nikolovski, I. Fildishevski, E. Minova, M. Mohamad Salmi, A. Md Nor, F. Elagili, M. Sainal, A. Zainudin, F. Hayati, R. Sriram, S. Subramaniam, J. Mah, S. Tan, A. Zakaria, Z. Zakaria, M. Wong, K. Hamdan, S. Dass, P. Andrejevic, J. Psaila, C. Cini, J. Schembri Higgins, M. Portelli, A. Majbar, A. Souadka, A. Benkabbou, S. Echiguer, M. El Hassouni, J. Konsten, F. Aarts, F. van Osch, T. Schok, E. Boerma, M. Martens, I. Poodt, K. van Dam, I. Bissett, C. Varghese, W. Xu, G. O’Brien, J. Penfold, M. Alvarez, R. He, A. Lin, P. Fagan, S. Farrant, S. Jury, J. Canton, C. Harmston, M. McGuinness, H. Witcomb‐Cahill, H. Boyes, A. Adeyeye, E. Afekahi, T. Chawla, U. Waqar, A. Fatimi, M. Arshad, A. Rehman, R. Aziz, S. Haider, A. Hai, S. Ahmed, M. Stanczak, M. Cunha, B. Mendes, I. Miguel, J. Rachadell, E. Amorim, C. Carneiro, R. Rocha, F. Almeida, R. Pera, J. Frazão, J. Costa Pereira, C. Costa Pereira, O. Oliveira, N. Gonçalves, E. Gonçalves, S. Fortuna Martins, H. Devesa, O. Teslyak, R. Barradas, S. Marisa Marques, A. Faustino, M. Almeida, D. Acosta, R. Quintanilha, P. Silva‐Vaz, H. Perez, R. Rainho, R. Monteiro, T. Neves, M. Yousif, A. Ahmed, A. Parvaiz, M. Abunada, A. Aleter, M. Dimofte, S. Morarasu, A. Musina, N. Velenciuc, S. Lunca, A. Yanishev, M. Bagrjancev, P. Zarubenko, A. Abelevich, A. Kokobelyan, A. Karachun, A. Olkina, T. Lankov, D. Samsonov, A. Petrov, S. Chowdhury, S. Alshahrani, J. Grosek, J. Košir, T. Košir Božič, A. Tomažič, J. Arredondo, J. Baixauli, C. Pastor, C. Sanchez, A. Alvarellos, V. Maderuelo Garcia, A. Huidobro Piriz, C. Suero Rodriguez, E. Castrillo Arconada, H. Ordas Macias, C. Martinez Perez, M. García Coret, C. Baez Burgos, C. Cifre Martinez, M. Tome Jimenez, R. García‐Domínguez, J. Cutillas‐Abellán, A. Fluixà‐Pelegrí, N. Ridaura‐Capellino, J. Seguí‐Gregori, S. Serra Pla, T. Labró, C. Soto, C. Gómez, P. Collera, J. Ocaña, J. Die, J. Fernandez‐Cebrian, P. Pastor, A. Garcia‐Chiloeches, A. Ballestero, L. Juez, J. Dziakova, J. Muguerza, D. Rivera Alonso, V. Catalan, M. Sajonia‐Coburgo, D. Moro‐Valdezate, V. Pla‐Marti, J. Martin‐Arevalo, A. Espi‐Macias, L. Perez‐Santiago, M. Carrasco, P. Parra, J. Muñoz, E. Peña, M. Ramírez, P. Tejedor, L. Jimenez, M. Labalde, P. Pelaez Torres, C. Nevado García, E. Ferrero Herrero, F. Garcia Borda, J. Bernal‐Sprekelsen, S. Gómez‐Abril, T. Torres, E. Martí, N. Borda Arrizabalaga, G. Elorza‐Echaniz, A. Andres‐Imaz, I. Aguirre‐Allende, M. Duque‐Mallen, C. Gracia‐Roche, M. Sanchez‐Fuentes, A. Martinez‐German, M. Santero‐Ramirez, M. Diez‐Alonso, M. Estaire Gómez, J. Valverde Mantecón, J. Martín Ramiro, E. Cagigal Ortega, M. Cancelas Felgueras, E. Colás‐Ruiz, J. Lliteras‐Jorge, M. Tasende‐Presedo, S. Baena‐Bradaschia, L. Lozano‐Salva, J. Gomez‐Rosado, J. Valdes‐Hernandez, F. DelRio‐Lafuente, J. Cintas‐Catena, A. Perez‐Sanchez, J. Abrisqueta, N. Ibáñez, B. Arencibia‐Pérez, C. Roque‐Castellano, E. Nogués‐Ramia, Y. Sosa‐Quesada, M. Artiles‐Armas, S. Gortázar de las Casas, I. Pascual Migueláñez, M. Álvarez Gallego, A. Gegundez Simón, B. Monje Vera, A. Minaya‐Bravo, A. Sanchez‐Gollarte, A. Galvan‐Perez, E. Gonzalez‐Gonzalez, S. Alonso, M. Jiménez, S. Salvans, M. Pascual, B. Montcusí, E. Viejo Martínez, J. Ripolles, M. de Fuenmayor Valera, C. Ortiz Johansson, E. Alvaro Cifuentes, E. Gutiérrez Cafranga, R. Escalera Pérez, W. Sánchez Bautista, J. Esteban Ramos, F. García Molina, L. Cristobal, M. Gomez, C. Cagigas, N. Suarez, G. Valero‐Navarro, E. Pellicer‐Franco, V. Soria‐Aledo, M. Mengual‐Ballester, J. Garcia‐Marin, A. Solís Peña, C. Petrola Chacón, M. Kraft Carré, F. Marinello, E. Espín Basany, B. De Andrés‐Asenjo, A. Romero, A. Vázquez, G. Cabezudo, J. Beltrán de Heredia, D. Wickramasinghe, S. Galal‐Eldin, H. Hamid, N. Hashim, M. Abdulmajeed, R. Elhadi, E. Abuobaida Bannaga, R. Hashim, F. Osman, H. Fadlalmola, A. Eltayeb, A. Ameen, S. Al Assaad, A. Papp, M. Pelczar, P. Buchwald, M. Lydrup, N. Azhar, T. Vedin, H. Jutesten, F. Ris, E. Liot, N. Buchs, L. Gialamas, A. Litchinko, B. Schiltz, C. Viehl, A. Müller, A. Lehnen, C. Regula, I. Fournier, E. Kalogiannis, S. Gussago, D. Chappalley, B. Guendil, R. Galli, R. Rosenberg, K. Roosen, M. Adamina, G. Peros, P. Müller, M. Giardini, T. Müller, M. von Strauss und Torney, M. Bolli, A. Polutak, S. Wullschleger, K. Herzog, S. Däster, S. Soysal, F. Haak, G. Hess, B. Wiesler, H. Al Houri, A. Alhouri, S. Jomaa, M. Daher, S. Abbas, M. Klib, A. Hammadieh, M. Ghandour, M. Chikh Salem, O. Danawer, M. Chaouch, K. Zouari, T. Kellil, E. Aytaç, C. Saracoglu, V. Özben, M. Gulmez, A. Mutlu, T. Olmez, A. Seker, A. Sozutek, K. Kaplan, S. Zenger, B. Gurbuz, U. Can, T. Yalti, D. Bugra, I. Gecim, M. Koc, C. Akyol, S. Ersoz, A. Erkek, Y. Altinel, S. Meric, K. Ozdogan, Y. Aktimur, A. Barcin, E. Balik, E. Ozoran, I. Ozata, D. Uymaz, C. Ulusoy, A. Demirel, S. Güçlü Mete, M. Atci, I. Cakcak, E. Aydoğdu, A. Goztepe, M. Ajredini, O. Ozkan, H. Ulgur, O. Duzgun, M. Kalin, E. Kirkan, A. Kebkalo, M. Boruta, V. Tyselskyi, Y. Tryliskyy, I. Kluzko, J. On, R. Shearer, M. Elhusseini, S. Coull, F. Carnegie, H. Joshi, J. Davies, C. Simillis, G. Theodoroleas, T. Hammond, J. Fairbanks, Z. Elliot, T. Khan, P. Nastro, L. Poynter, S. Nikolaou, R. Bhardwaj, J. Adamek, B. Stubbs, G. Neelankavil Davis, J. Eid, Z. Naumowicz, M. Gupta, J. Sebastian, S. Mangam, J. Evans, G. Preziosi, R. Koshy, R. Harshen, M. Badawi, A. Rahman, I. Rakhimov, N. Cruikshank, J. Kynaston, M. Yule, F. Reilly, K. Marimuthu, A. John, S. Bhanderi, R. Woods, L. Taylor, M. Dhruva Rao, M. Afridi, I. Elnagar, S. Arnold, F. Di Fabio, A. Venkatasubramaniam, C. Yao, E. Anand, A. Myers, Y. Mohsen, A. Prabhudesai, A. Slesser, S. Mallappa, G. Bond‐Smith, T. Fung, S. Kok, F. Runau, B. Singh, S. Khan, A. Eltweri, J. Wolff, K. Boyle, P. Sarmah, I. Kais, H. Merriman, S. Gurjar, M. Al‐Ani, N. Ul‐Ain, H. Tabasi, B. Keeler, F. Dixon, M. Rana, J. Khan, R. Harvitkar, S. Stefan, R. Duhoky, R. Bethune, H. Lederhuber, T. Cheung, E. Matthews, K. Altaf, N. Pang, C. Simms, G. Titley‐Wilson, M. Thaha, A. Minicozzi, H. Patel, A. Taha, G. Omar, M. Riad, C. Magee, S. Small, L. Convie, J. Ong, C. Rossborough, N. Chandratreya, J. Noronha, M. Alshibshoubi, E. Dean, J. Thompson

**Keywords:** anastomotic leak, benchmarking, colorectal resection, enhanced recovery programme, ESCP audit, minimally invasive surgery, perioperative management, postoperative morbidity, prospective multicentre audit

## Abstract

**Introduction:**

Benchmarking colorectal surgery outcomes informs quality improvement. The ESCP CORREA 2022 snapshot audit aimed to assess contemporary colorectal resection practices and short‐term outcomes across European countries and beyond.

**Methods:**

An international prospective multicentre audit was conducted in which adults undergoing elective or emergency colorectal resection during a 6‐week period (January–April 2022) at participating hospitals were included. Data on patient demographics, indications, surgical approach (open, laparoscopic or robotic) and 30‐day postoperative outcomes (complications, reoperation and mortality) were collected for analysis. The outcomes were analysed and compared with those of previous audits to identify trends in colorectal surgery.

**Results:**

The study enrolled 3521 patients (56.8% men) from 216 hospitals across 53 countries. In 72.2% of the cases, the indication for resection was malignancy, followed by diverticular disease in 9.0%, Crohn's disease in 3.7% and ulcerative colitis in 2.3% of the cases. Of the surgeries, 74.4% were elective. Minimally invasive surgery was performed in 55.2% of the cases (48.7% laparoscopic and 6.5% robotic). Primary anastomosis was performed in 90.3% of the patients. The 30‐day anastomotic leak rate was 7.96%; in malignant and benign diseases, the leak rates were 7.3% and 10.2%, respectively. The leak rates for right, left, anterior rectal resection, pouch and subtotal colectomy were 6.9%, 7.7%, 9.7%, 16.0% and 11.8%, respectively. In the multivariable analysis, the risk factors for leakage included male sex (9.3% vs. 6.3%, OR = 0.69, 95% CI 0.51–0.95, *p* = 0.023) and emergency surgery (11.4% vs. 7.1%, OR = 1.58, 95% CI 1.10–2.27, *p* = 0.013). Thirty‐day mortality was 2.38%.

**Conclusions:**

This large international audit provides the status of the management of colorectal surgery. This shows that minimally invasive techniques are widely adopted, and 30‐day mortality is low; however, anastomotic leak rates remain persistently high. These findings highlight the ongoing need for targeted research and quality‐improvement initiatives to reduce anastomotic failure and improve outcomes of colorectal surgery.

**What does this paper add to the literature?**

European Society of Coloproctology CORREA 2022 audit provides updated real‐world benchmarks, enrolling 3521 patients from 216 hospitals in 53 countries, extending 2015/2017 audits. MIS predominated (55.2%; 48.7% laparoscopic, 6.5% robotic); mortality was 2.38%, yet leaks persisted (7.96%), linked to male sex (9.3% vs. 6.3%) and emergency surgery (11.4% vs. 7.1%), driving research and quality improvement.

## INTRODUCTION

Colorectal cancer (CRC) represents the third cause of cancer‐related mortality [1], with surgery being the primary curative treatment [2]. Colorectal resections are also performed for benign diseases, including complicated diverticulitis and inflammatory bowel disease, both of which account for significant colorectal surgical volume [3]. A recent nationwide analysis reported that nearly one‐quarter of left‐sided colectomies were performed for diverticulitis [4].

Over the past few decades, advances in surgical techniques, such as minimally invasive surgery, perioperative care (enhanced recovery after surgery protocols), imaging and intensive care, have improved the outcomes of colorectal surgeries [5]. However, colorectal resection is still associated with considerable postoperative morbidity, and anastomotic leak remains one of the most severe complications, with an incidence rate of approximately 8.1% in a recent series [6, 7]. In the 2015 ESCP audit of right colectomies, 30‐day mortality increased from 1.6% in patients without leaks to 10.7% in those with leaks [8].

Marked international heterogeneity in colorectal surgical practices and outcomes complicates systematic quality improvement. In the GlobalSurg 3 study, 30‐day mortality after CRC surgery was approximately four‐fold higher in low‐income settings and approximately two‐fold higher in lower‐middle‐income settings than in high‐income countries [9]. While case mix and disease presentation contribute, differences are driven mainly by disparities in perioperative care, infrastructure and the ability to identify complications. These findings highlight the need for contemporary benchmarking to identify best practices and drive global quality improvements. Multicentre audits have proven effective for evaluating practice and outcomes in colorectal surgery. The European Society of Coloproctology (ESCP) has coordinated snapshot audits across countries, generating large contemporary datasets [6, 10, 11, 12, 13].

The ESCP 2022 COloRectal Resection Audit (CORREA) aimed to enrol all adult patients undergoing elective or emergency colorectal resection across participating centres to characterise contemporary surgical practice, perioperative management and short‐term (30‐day) outcomes in diverse healthcare settings. A further objective was to generate up‐to‐date benchmarks that inform the ESCP quality‐improvement initiatives and identify priorities for future research to enhance patient care.

## METHODS

### Study design and setting

The ESCP CORREA 2022 study was a prospective, multicentre cohort study conducted as an international snapshot audit [14]. This methodology allowed the generation of large contemporaneous datasets that reflect real‐world practices and enable comparisons across units and regions. The CORREA audit was internationally accessible to hospitals performing colorectal surgery and was organised by the ESCP Cohort Studies Working Group [14]. Recruitment was conducted through ESCP dissemination channels, targeting centres that had previously participated in ESCP audits, as well as all ESCP members; participating centres were self‐selected.

### Patient inclusion and data collection

Each participating site identified all consecutive patients during a self‐selected 6‐week interval, starting between the 17th of January 2022 and 30th of April 2022. This rolling start window allowed flexibility for centres to commence data collection at any time in the first quarter of 2022 (6 weeks per site). The inclusion criteria were adults aged ≥18 years who underwent colorectal resection (defined as excision of any part of the colon and/or rectum) during the study period. This included both elective and emergency surgeries and any indication (neoplastic (malignant and benign), inflammatory (diverticular disease, inflammatory bowel disease) and trauma). Resections with or without anastomosis (i.e., primary anastomosis or end stoma formation) were included. There were no specific exclusion criteria, aside from age < 18 years and procedures not involving resection of the colon/rectum (stoma closure, stricturoplasty with or without colorectal resection, pelvic exenteration, more than one anastomosis or cytoreductive surgery).

Local investigators prospectively collected data from each site using a standardised electronic case report form in the secure online REDCap database hosted by the University of Birmingham. To ensure data quality, all fields had predefined options and range checks. Anonymised patient identifiers were used to verify completeness and follow‐up. No identifiable patient information was collected. A collaborative authorship model was used, with all investigators contributing data included as PubMed‐citable co‐authors under the group name, in line with other publications from this group (references to previous audit publications).

### Variables and definitions

Patient variables, including age, sex, body mass index (BMI), American Society of Anaesthesiologists (ASA) physical classification (categorised as 1–2 vs. ≥3), and comorbidities (including cardiovascular disease and diabetes), were also recorded. Obesity was classified according to the World Health Organisation criteria [15]. Disease variables: Indications for surgery were categorised (CRC, benign polyps, inflammatory bowel disease, diverticular disease and others). Specific details were collected for Crohn's disease, ulcerative colitis and cancer (including histopathological results) [14]. Operative variables included the urgency of surgery (elective, expedited – within 2 weeks of decision, or emergency – within 24 hours of decision), type of resection (right hemicolectomy, left/sigmoid colectomy, anterior resection, abdominoperineal resection or subtotal/total colectomy) and stoma formation. The surgical approach was defined as the approach at the start of the operation and categorised as open, laparoscopic or robotic. Conversions from the minimally invasive to the open approach were also monitored. For anastomoses, the technique was documented as hand‐sewn versus stapled and whether diversion (proximal protective stoma) was created. A structured form for the anastomotic technique was included, noting the configuration (end‐to‐end or side‐to‐side) and intraoperative leak testing to enable later analysis of the technique on the outcomes. The surgeon's specialisation (colorectal specialist vs. general surgeon) was also recorded. Outcomes: 30‐day postoperative complications, reoperations, enhanced recovery programme details, hospital stay and 30‐day readmission rate. Surgical site infections were defined according to the Centers for Disease Control and Prevention criteria [16].

### Data analysis

At the end of the data collection (database lock on 11th of July2022), the steering group cleaned and analysed the combined dataset. Summary statistics were used to describe the cohort. Categorical variables are presented as frequencies with corresponding percentages, whereas continuous variables are described using either the mean ± standard deviation or the median with the interquartile range, as appropriate. Chi‐square tests were used to assess the differences between groups. Multivariable logistic regression was employed to identify independent risk factors for anastomotic leakage, with variables selected based on clinical relevance and univariate significance. A two‐sided *p* < 0.05 was considered to be statistically significant. This study followed the STROBE guidelines for observational studies.

### Ethical considerations

Formal research ethics committee approvals were obtained where needed, although this was not necessary in many participating centres, as this was viewed as an audit of routine practice with anonymised data collection. Each collaborating investigator/unit was responsible for complying with local audit/governance requirements. The audit protocol was approved by the ESCP Research Committee and circulated among all collaborators. According to the local policy of the participating hospitals, patient consent was obtained for the inclusion of deidentified data. The study was registered with clinical audit registries, as required by individual hospitals.

## RESULTS

### Patient characteristics

**FIGURE 1:**
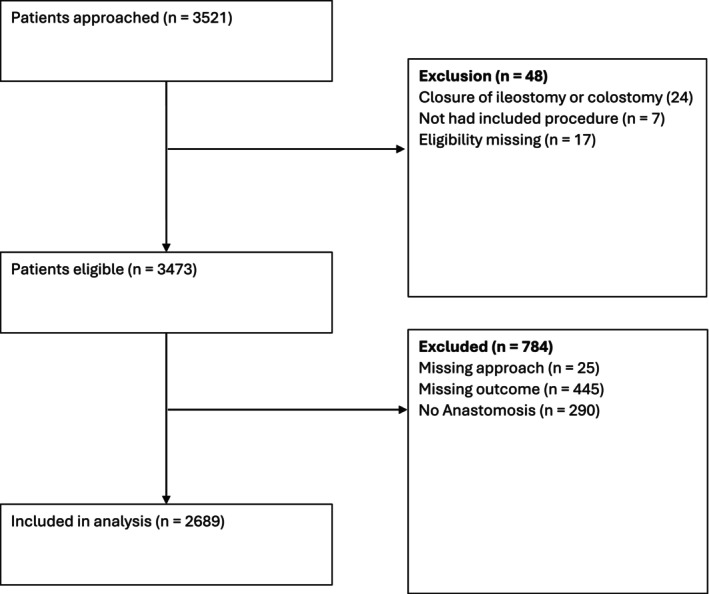
CONSORT diagram of the anastomotic leakage study.

The audit included 3521 patients who underwent colorectal resection at 216 hospitals across 53 countries (Figure 1, Table 1). The final cohort comprised academic teaching hospitals, district general hospitals and tertiary referral centres across Europe, Africa, Asia, the Americas (South America and the Caribbean) and Australasia. The cohort was predominantly middle‐aged and elderly, with 36.5% aged 55–70 years and 39.9% above 70 years. Males comprised 56.8%, and BMI distribution showed 28.7% obese, 30.1% overweight and 11.4% underweight. Most patients (64.6%) were low risk (ASA grade I–II). Comorbidities included ischaemic heart disease or stroke in 11.7% and diabetes in 17.7%. Regarding smoking, 60.1% never smoked, 14.5% were current smokers and the rest were former smokers. No significant differences existed between surgical groups in cardiovascular comorbidities, diabetes or smoking status (*p* = 0.200, *p* = 0.064, *p* = 0.096). Malignancy was the main indication (72.2%), followed by diverticular disease (9.0%), Crohn's disease (3.7%) and other conditions. Preoperative neoadjuvant treatment varied by approach (*p* < 0.001), with 27.2% robotic, 14.3% laparoscopic and 11.6% open surgery patients receiving therapy. Short‐ and long‐course chemoradiotherapy rates were similar between groups.

**TABLE 1:** Patient characteristics correlated with the surgical approach.

Variable	Levels	Open (*N* = 1246)	Robotic (*N* = 224)	Laparoscopic (*N* = 1680)	Robotic‐open (*N* = 10)	Lap – open (*N* = 288)	Total (*N* = 3448)	*p*
Age	Less than 55	324 (26.0)	39 (17.4)	391 (23.3)	2 (20.0)	57 (19.8)	813 (23.6)	<0.001
	Between 55 and 70	423 (33.9)	103 (46.0)	630 (37.5)	3 (30.0)	99 (34.4)	1258 (36.5)	
	Between 70 and 80	306 (24.6)	59 (26.3)	474 (28.2)	2 (20.0)	88 (30.6)	929 (26.9)	
	Over 80	193 (15.5)	23 (10.3)	185 (11.0)	3 (30.0)	44 (15.3)	448 (13.0)	
	(Missing)	0 (0.0)	0 (0.0)	0 (0.0)	0 (0.0)	0 (0.0)	0 (0.0)	
Sex	Male	709 (56.9)	146 (65.2)	923 (54.9)	6 (60.0)	176 (61.1)	1960 (56.8)	0. 0225
	Female	537 (43.1)	78 (34.8)	757 (45.1)	4 (40.0)	112 (38.9)	1488 (43.2)	
	(Missing)	0 (0.0)	0 (0.0)	0 (0.0)	0 (0.0)	0 (0.0)	0 (0.0)	
ASA class	Low risk (ASA 1 & 2)	755 (60.6)	155 (69.2)	1128 (67.1)	6 (60.0)	185 (64.2)	2229 (64.6)	0.002
	High risk (ASA 3 and above)	491 (39.4)	69 (30.8)	552 (32.9)	4 (40.0)	103 (35.8)	1219 (35.4)	
	(Missing)	0 (0.0)	0 (0.0)	0 (0.0)	0 (0.0)	0 (0.0)	0 (0.0)	
BMI	Normal weight	350 (28.1)	84 (37.5)	520 (31.0)	2 (20.0)	74 (25.7)	1030 (29.9)	<0.001
	Underweight	172 (13.8)	17 (7.6)	174 (10.4)	0 (0.0)	30 (10.4)	393 (11.4)	
	Overweight	328 (26.3)	83 (37.1)	541 (32.2)	4 (40.0)	81 (28.1)	1037 (30.1)	
	Obese	396 (31.8)	40 (17.9)	445 (26.5)	4 (40.0)	103 (35.8)	988 (28.7)	
	(Missing)	0 (0.0)	0 (0.0)	0 (0.0)	0 (0.0)	0 (0.0)	0 (0.0)	
History of IHD/stroke	Yes	167 (13.4)	24 (10.7)	177 (10.5)	1 (10.0)	33 (11.5)	402 (11.7)	0.200
	No	1079 (86.6)	200 (89.3)	1503 (89.5)	9 (90.0)	255 (88.5)	3046 (88.3)	
	(Missing)	0 (0.0)	0 (0.0)	0 (0.0)	0 (0.0)	0 (0.0)	0 (0.0)	
History of diabetes mellitus	Yes	241 (19.3)	33 (14.7)	273 (16.2)	2 (20.0)	60 (20.8)	609 (17.7)	0.064
	No	1004 (80.6)	191 (85.3)	1407 (83.8)	8 (80.0)	228 (79.2)	2838 (82.3)	
	(Missing)	1 (0.1)	0 (0.0)	0 (0.0)	0 (0.0)	0 (0.0)	1 (0.0)	
Smoking	Never	751 (60.3)	120 (53.6)	1027 (61.1)	4 (40.0)	170 (59.0)	2072 (60.1)	0.069
	Current smoker	181 (14.5)	35 (15.6)	239 (14.2)	1 (10.0)	45 (15.6)	501 (14.5)	
	Ex‐smoker (<6 weeks)	60 (4.8)	6 (2.7)	51 (3.0)	1 (10.0)	9 (3.1)	127 (3.7)	
	Ex‐smoker (>6 weeks)	251 (20.1)	63 (28.1)	359 (21.4)	4 (40.0)	63 (21.9)	740 (21.5)	
	(Missing)	3 (0.2)	0 (0.0)	4 (0.2)	0 (0.0)	1 (0.3)	8 (0.2)	
Indication	Benign polyp	14 (1.1)	10 (4.5)	59 (3.5)	0 (0.0)	7 (2.4)	90 (2.6)	<0.001
	Crohn's disease	46 (3.7)	2 (0.9)	67 (4.0)	1 (10.0)	13 (4.5)	129 (3.7)	
	Diverticular disease	120 (9.6)	12 (5.4)	145 (8.6)	1 (10.0)	31 (10.8)	309 (9.0)	
	Malignancy (cancer)	809 (64.9)	185 (82.6)	1297 (77.2)	8 (80.0)	192 (66.7)	2491 (72.2)	
	Adenomatous polyposis	9 (0.7)	8 (3.6)	20 (1.2)	0 (0.0)	4 (1.4)	41 (1.2)	
	Ulcerative colitis	25 (2.0)	5 (2.2)	43 (2.6)	0 (0.0)	5 (1.7)	78 (2.3)	
	Ischaemic colitis	39 (3.1)	0 (0.0)	0 (0.0)	0 (0.0)	6 (2.1)	45 (1.3)	
	Microscopic colitis	5 (0.4)	0 (0.0)	0 (0.0)	0 (0.0)	0 (0.0)	5 (0.1)	
	Trauma	19 (1.5)	0 (0.0)	0 (0.0)	0 (0.0)	2 (0.7)	21 (0.6)	
	Other	160 (12.8)	2 (0.9)	49 (2.9)	0 (0.0)	28 (9.7)	239 (6.9)	
	(Missing)	0 (0.0)	0 (0.0)	0 (0.0)	0 (0.0)	0 (0.0)	0 (0.0)	
Urgency	Planned	692 (55.5)	208 (92.9)	1452 (86.4)	10 (100.0)	205 (71.2)	2567 (74.4)	<0.001
	emergency/expedited	553 (44.4)	16 (7.1)	228 (13.6)	0 (0.0)	83 (28.8)	880 (25.5)	
	(Missing)	1 (0.1)	0 (0.0)	0 (0.0)	0 (0.0)	0 (0.0)	1 (0.0)	
Resection procedure	Ileocaecal resection	58 (4.7)	7 (3.1)	72 (4.3)	1 (10.0)	16 (5.6)	154 (4.5)	<0.001
	Right hemicolectomy	296 (23.8)	44 (19.6)	463 (27.6)	2 (20.0)	72 (25.0)	877 (25.4)	
	Extended right hemicolectomy	72 (5.8)	5 (2.2)	81 (4.8)	0 (0.0)	22 (7.6)	180 (5.2)	
	Left hemicolectomy	111 (8.9)	17 (7.6)	131 (7.8)	0 (0.0)	30 (10.4)	289 (8.4)	
	Sigmoid colectomy	175 (14.0)	35 (15.6)	317 (18.9)	1 (10.0)	46 (16.0)	574 (16.6)	
	Hartmann procedure	131 (10.5)	2 (0.9)	33 (2.0)	1 (10.0)	19 (6.6)	186 (5.4)	
	Subtotal colectomy	82 (6.6)	0 (0.0)	45 (2.7)	0 (0.0)	10 (3.5)	137 (4.0)	
	Anterior resection	166 (13.3)	86 (38.4)	395 (23.5)	4 (40.0)	53 (18.4)	704 (20.4)	
	Transverse colon resection	32 (2.6)	1 (0.4)	12 (0.7)	0 (0.0)	3 (1.0)	48 (1.4)	
	APER	61 (4.9)	21 (9.4)	83 (4.9)	1 (10.0)	11 (3.8)	177 (5.1)	
	Restorative proctocolectomy	12 (1.0)	1 (0.4)	14 (0.8)	0 (0.0)	2 (0.7)	29 (0.8)	
	Proctectomy	46 (3.7)	4 (1.8)	25 (1.5)	0 (0.0)	1 (0.3)	76 (2.2)	
	Ileoanal pouch procedure	2 (0.2)	1 (0.4)	8 (0.5)	0 (0.0)	3 (1.0)	14 (0.4)	
	(Missing)	2 (0.2)	0 (0.0)	1 (0.1)	0 (0.0)	0 (0.0)	3 (0.1)	
Primary surgeon in charge	Colorectal trainee	37 (3.0)	13 (5.8)	106 (6.3)	2 (20.0)	16 (5.6)	174 (5.0)	<0.001
	Colorectal consultant surgeon	619 (49.7)	176 (78.6)	1231 (73.3)	5 (50.0)	196 (68.1)	2227 (64.6)	
	General surgery trainee	119 (9.6)	1 (0.4)	60 (3.6)	0 (0.0)	17 (5.9)	197 (5.7)	
	General consultant surgeon	471 (37.8)	34 (15.2)	283 (16.8)	3 (30.0)	59 (20.5)	850 (24.7)	
	(Missing)	0 (0.0)	0 (0.0)	0 (0.0)	0 (0.0)	0 (0.0)	0 (0.0)	
Malignancy (cancer)	No	422 (33.9)	37 (16.5)	419 (24.9)	4 (40.0)	96 (33.3)	978 (28.4)	<0.001
	Yes	814 (65.3)	182 (81.2)	1244 (74.0)	6 (60.0)	190 (66.0)	2436 (70.6)	
	(Missing)	10 (0.8)	5 (2.2)	17 (1.0)	0 (0.0)	2 (0.7)	34 (1.0)	
Biopsy verified	No	176 (14.1)	7 (3.1)	76 (4.5)	0 (0.0)	15 (5.2)	274 (7.9)	<0.001
	Yes	633 (50.8)	178 (79.5)	1221 (72.7)	8 (80.0)	177 (61.5)	2217 (64.3)	
	(Missing)	437 (35.1)	39 (17.4)	383 (22.8)	2 (20.0)	96 (33.3)	957 (27.8)	
Initial pre‐treatment T stage	T1	62 (5.0)	17 (7.6)	172 (10.2)	1 (10.0)	19 (6.6)	271 (7.9)	<0.001
	T2	178 (14.3)	51 (22.8)	336 (20.0)	3 (30.0)	39 (13.5)	607 (17.6)	
	T3	350 (28.1)	77 (34.4)	578 (34.4)	1 (10.0)	84 (29.2)	1090 (31.6)	
	T4	172 (13.8)	27 (12.1)	142 (8.5)	2 (20.0)	41 (14.2)	384 (11.1)	
	(Missing)	484 (38.8)	52 (23.2)	452 (26.9)	3 (30.0)	105 (36.5)	1096 (31.8)	
Initial pre‐treatment M stage	M0	670 (53.8)	163 (72.8)	1183 (70.4)	8 (80.0)	171 (59.4)	2195 (63.7)	<0.001
	M1	107 (8.6)	15 (6.7)	84 (5.0)	0 (0.0)	18 (6.2)	224 (6.5)	
	(Missing)	469 (37.6)	46 (20.5)	413 (24.6)	2 (20.0)	99 (34.4)	1029 (29.8)	
Initial pre‐treatment N stage	N0	364 (29.2)	94 (42.0)	725 (43.2)	4 (40.0)	100 (34.7)	1287 (37.3)	<0.001
	N1	267 (21.4)	58 (25.9)	376 (22.4)	0 (0.0)	62 (21.5)	763 (22.1)	
	N2	132 (10.6)	25 (11.2)	143 (8.5)	3 (30.0)	20 (6.9)	323 (9.4)	
	(Missing)	483 (38.8)	47 (21.0)	436 (26.0)	3 (30.0)	106 (36.8)	1075 (31.2)	
Threatened (<2 mm) CRM on MRI	No	614 (49.3)	131 (58.5)	1090 (64.9)	6 (60.0)	157 (54.5)	1998 (57.9)	<0.001
	Yes	120 (9.6)	35 (15.6)	127 (7.6)	1 (10.0)	27 (9.4)	310 (9.0)	
	(Missing)	512 (41.1)	58 (25.9)	463 (27.6)	3 (30.0)	104 (36.1)	1140 (33.1)	
Preoperative Neoadjuvant therapy	No	664 (53.3)	124 (55.4)	1057 (62.9)	4 (40.0)	165 (57.3)	2014 (58.4)	<0.001
	Yes	144 (11.6)	61 (27.2)	240 (14.3)	4 (40.0)	27 (9.4)	476 (13.8)	
	(Missing)	438 (35.2)	39 (17.4)	383 (22.8)	2 (20.0)	96 (33.3)	958 (27.8)	
Chemotherapy Only	No	88 (7.1)	53 (23.7)	205 (12.2)	3 (30.0)	22 (7.6)	371 (10.8)	<0.001
	Yes	56 (4.5)	8 (3.6)	35 (2.1)	1 (10.0)	5 (1.7)	105 (3.0)	
	(Missing)	1102 (88.4)	163 (72.8)	1440 (85.7)	6 (60.0)	261 (90.6)	2972 (86.2)	
SCRT: Short‐course radiotherapy	No	51 (4.1)	32 (14.3)	131 (7.8)	2 (20.0)	14 (4.9)	230 (6.7)	0.895
	Yes	37 (3.0)	21 (9.4)	73 (4.3)	1 (10.0)	8 (2.8)	140 (4.1)	
	(Missing)	1158 (92.9)	171 (76.3)	1476 (87.9)	7 (70.0)	266 (92.4)	3078 (89.3)	
Long‐course chemoradiotherapy	No	37 (3.0)	15 (6.7)	68 (4.0)	1 (10.0)	7 (2.4)	128 (3.7)	0.526
	Yes	52 (4.2)	38 (17.0)	137 (8.2)	2 (20.0)	15 (5.2)	244 (7.1)	
	(Missing)	1157 (92.9)	171 (76.3)	1475 (87.8)	7 (70.0)	266 (92.4)	3076 (89.2)	
Irritable Bowel Disease	0	1147 (92.1)	213 (95.1)	1541 (91.7)	9 (90.0)	265 (92.0)	3175 (92.1)	0.122
	1	81 (6.5)	6 (2.7)	116 (6.9)	1 (10.0)	19 (6.6)	223 (6.5)	
	(Missing)	18 (1.4)	5 (2.2)	23 (1.4)	0 (0.0)	4 (1.4)	50 (1.5)	
Systemic steroids	No	49 (3.9)	6 (2.7)	75 (4.5)	1 (10.0)	15 (5.2)	146 (4.2)	0.715
	Yes	22 (1.8)	1 (0.4)	33 (2.0)	0 (0.0)	3 (1.0)	59 (1.7)	
	(Missing)	1175 (94.3)	217 (96.9)	1572 (93.6)	9 (90.0)	270 (93.8)	3243 (94.1)	
Immuno‐modulators	No	57 (4.6)	6 (2.7)	85 (5.1)	1 (10.0)	16 (5.6)	165 (4.8)	0.890
	Yes	14 (1.1)	1 (0.4)	23 (1.4)	0 (0.0)	2 (0.7)	40 (1.2)	
	(Missing)	1175 (94.3)	217 (96.9)	1572 (93.6)	9 (90.0)	270 (93.8)	3243 (94.1)	
Biological agent	No	54 (4.3)	7 (3.1)	76 (4.5)	0 (0.0)	12 (4.2)	149 (4.3)	0.172
	Yes	17 (1.4)	0 (0.0)	32 (1.9)	1 (10.0)	6 (2.1)	56 (1.6)	
	(Missing)	1175 (94.3)	217 (96.9)	1572 (93.6)	9 (90.0)	270 (93.8)	3243 (94.1)	
Preoperative antibiotic	No	45 (3.6)	5 (2.2)	83 (4.9)	1 (10.0)	15 (5.2)	149 (4.3)	0.234
	Yes	26 (2.1)	2 (0.9)	25 (1.5)	0 (0.0)	3 (1.0)	56 (1.6)	
	(Missing)	1175 (94.3)	217 (96.9)	1572 (93.6)	9 (90.0)	270 (93.8)	3243 (94.1)	
Steroid stress dose	No	64 (5.1)	7 (3.1)	94 (5.6)	1 (10.0)	17 (5.9)	183 (5.3)	0.722
	Yes	7 (0.6)	0 (0.0)	15 (0.9)	0 (0.0)	1 (0.3)	23 (0.7)	
	(Missing)	1175 (94.3)	217 (96.9)	1571 (93.5)	9 (90.0)	270 (93.8)	3242 (94.0)	
Enteric fistula	No	51 (4.1)	7 (3.1)	98 (5.8)	1 (10.0)	13 (4.5)	170 (4.9)	0.013
	Yes	20 (1.6)	0 (0.0)	12 (0.7)	0 (0.0)	5 (1.7)	37 (1.1)	
	(Missing)	1175 (94.3)	217 (96.9)	1570 (93.5)	9 (90.0)	270 (93.8)	3241 (94.0)	
Pre‐existing operative abscess	No	56 (4.5)	6 (2.7)	100 (6.0)	1 (10.0)	15 (5.2)	178 (5.2)	0.102
	Yes	15 (1.2)	1 (0.4)	9 (0.5)	0 (0.0)	3 (1.0)	28 (0.8)	
	(Missing)	1175 (94.3)	217 (96.9)	1571 (93.5)	9 (90.0)	270 (93.8)	3242 (94.0)	
Anastomosis	Handsewn	262 (21.0)	17 (7.6)	200 (11.9)	1 (10.0)	58 (20.1)	538 (15.6)	<0.001
	Stapled	553 (44.4)	172 (76.8)	1272 (75.7)	7 (70.0)	171 (59.4)	2175 (63.1)	
	None	189 (15.2)	8 (3.6)	65 (3.9)	0 (0.0)	28 (9.7)	290 (8.4)	
	(Missing)	242 (19.4)	27 (12.1)	143 (8.5)	2 (20.0)	31 (10.8)	445 (12.9)	

### Surgical approach

Half of colorectal resections used minimally invasive surgery, with 48.7% (*n* = 1680) laparoscopic and 6.5% (*n* = 224) robotic (Table 2). Open surgery comprised 36.1% (*n* = 1246) of the cases, while conversion rates to open surgery were 14.6% for laparoscopic and 4.3% for robotic approaches, representing 8.4% and 0.3% of the total cases, respectively.

**TABLE 2:** Postoperative outcomes correlated with the surgical approach.

Variable	levels	Open	Robotic	Laparoscopic	Robotic‐open	Lap – open	Total	*p*
Length of postoperative stay	Median (IQR)	8.0 (6.0–14.0)	5.0 (4.0–7.0)	6.0 (4.0–8.0)	13.0 (8.5–18.0)	7.0 (6.0–12.0)	7.0 (5.0–10.0)	<0.001
Clavien–Dindo grade of the worst complication (up to 30 days post index surgery)	None	404 (32.4)	131 (58.5)	971 (57.8)	3 (30.0)	113 (39.2)	1622 (47.0)	<0.001
	Grade I	372 (29.9)	41 (18.3)	375 (22.3)	1 (10.0)	82 (28.5)	871 (25.3)	
	Grade II	226 (18.1)	26 (11.6)	181 (10.8)	5 (50.0)	52 (18.1)	490 (14.2)	
	Grade III a	51 (4.1)	9 (4.0)	31 (1.8)	0 (0.0)	11 (3.8)	102 (3.0)	
	Grade III b	81 (6.5)	10 (4.5)	80 (4.8)	1 (10.0)	15 (5.2)	187 (5.4)	
	Grade IV a	19 (1.5)	1 (0.4)	12 (0.7)	0 (0.0)	2 (0.7)	34 (1.0)	
	Grade IV b	17 (1.4)	0 (0.0)	4 (0.2)	0 (0.0)	3 (1.0)	24 (0.7)	
	Grade V	66 (5.3)	1 (0.4)	9 (0.5)	0 (0.0)	8 (2.8)	84 (2.4)	
	(Missing)	10 (0.8)	5 (2.2)	17 (1.0)	0 (0.0)	2 (0.7)	34 (1.0)	
Leak	No	731 (58.7)	170 (75.9)	1360 (81.0)	7 (70.0)	207 (71.9)	2475 (71.8)	0.126
	Yes	77 (6.2)	14 (6.2)	100 (6.0)	1 (10.0)	22 (7.6)	214 (6.2)	
	(Missing)	438 (35.2)	40 (17.9)	220 (13.1)	2 (20.0)	59 (20.5)	759 (22.0)	
Leak proven	No	751 (60.3)	174 (77.7)	1382 (82.3)	7 (70.0)	219 (76.0)	2533 (73.5)	0.271
	Yes	57 (4.6)	10 (4.5)	78 (4.6)	1 (10.0)	10 (3.5)	156 (4.5)	
	(Missing)	438 (35.2)	40 (17.9)	220 (13.1)	2 (20.0)	59 (20.5)	759 (22.0)	
Surgical site infection	No	577 (46.3)	71 (31.7)	584 (34.8)	5 (50.0)	129 (44.8)	1366 (39.6)	<0.001
	Yes	256 (20.5)	17 (7.6)	111 (6.6)	2 (20.0)	45 (15.6)	431 (12.5)	
	(Missing)	413 (33.1)	136 (60.7)	985 (58.6)	3 (30.0)	114 (39.6)	1651 (47.9)	
Was the patient readmitted within 30 days of the index surgery?	No	1136 (91.2)	207 (92.4)	1573 (93.6)	10 (100.0)	266 (92.4)	3192 (92.6)	0.044
	Yes	100 (8.0)	11 (4.9)	89 (5.3)	0 (0.0)	20 (6.9)	220 (6.4)	
	(Missing)	10 (0.8)	6 (2.7)	18 (1.1)	0 (0.0)	2 (0.7)	36 (1.0)	
Did the patient undergo an additional operation within 30 day of the index surge	No	1097 (88.0)	211 (94.2)	1563 (93.0)	9 (90.0)	260 (90.3)	3140 (91.1)	<0.001
	Yes	139 (11.2)	8 (3.6)	100 (6.0)	1 (10.0)	26 (9.0)	274 (7.9)	
	(Missing)	10 (0.8)	5 (2.2)	17 (1.0)	0 (0.0)	2 (0.7)	34 (1.0)	

The analysis showed correlations between surgical approach and patient demographics. Robotic and laparoscopic surgeries were less common in patients over 80 years (10.3% and 11.0%) compared to open surgery (15.5%, *p* < 0.001) and had fewer high‐risk ASA III‐IV patients (30.8%, 32.9% and 39.4% respectively; *p* = 0.005). Obese patients underwent fewer robotic and laparoscopic surgeries than non‐obese patients (17.9% vs. 26.5% vs. 31.8% for open surgery, *p* < 0.001). Male patients were more prevalent in robotic procedures (65.2%) than in laparoscopic and open procedures (54.9% and 56.9%; *p* = 0.028).

Elective surgery comprised 74.4% of cases, while 25.5% were emergencies. Preoperative characteristics varied significantly by surgical approach, with elective and malignant conditions favouring minimally invasive techniques. Emergency procedures were more common in open surgery (44.4%) compared to laparoscopic (13.6%) and robotic surgeries (7.1%) (*p* < 0.001). Malignant resections were predominant in robotic surgery (82.6%) versus laparoscopic (77.2%) and open surgery (64.9%) (*p* < 0.001).

Right hemicolectomy (25.4%), anterior resection (20.4%) and sigmoid colectomy (16.6%) were the most common surgeries. Right hemicolectomy was performed in 33.8%, 52.8%, 5.0% and 8.4% of cases using open, laparoscopic, robotic and converted approaches, respectively (*p* < 0.001). Robotic surgery focused on rectal cancer, with anterior resections comprising 38.4% of robotic cases, higher than open (13.3%) and laparoscopic (23.6%) approaches (*p* < 0.001). Robotic abdominoperineal resection (9.4%) exceeded open surgery (4.9%). Open surgery dominated Hartmann's procedures (10.5% vs. 2.0% laparoscopic, 0.9% robotic) and subtotal colectomies (6.6% vs. 2.7% laparoscopic, 0% robotic), reflecting its use in urgent scenarios.

Minimally invasive procedures were performed by colorectal specialists, while open procedures were done by general surgeons (*p* < 0.001). Colorectal consultants led 78.6% of robotic and 73.3% of laparoscopic operations, compared to 49.7% in open surgery. General surgery consultants performed 37.8% of open cases versus 15.2% of robotic and 16.8% of laparoscopic surgeries, indicating that specialty training correlated with minimally invasive approaches.

### Postoperative outcomes

Hospital stays were shorter with minimally invasive approaches: median 5 days (IQR 4.0–7.0) for robotic and 6 days (4.0–8.0) for laparoscopic versus 8 days (6.0–14.0) for open surgeries (*p* < 0.001). Conversion cases had longer stays (13 days robotic to open, 7 days lap to open), reflecting increased recovery time.

Minimally invasive surgery shows lower postoperative morbidity. Over half of robotic (58.5%) and laparoscopic (57.8%) patients had no 30‐day complications versus 32.4% of open surgery patients (*p* < 0.001). Severe complications (Clavien–Dindo grade III–V) occurred more after open resection. The 30‐day mortality was higher in the open group (5.3% (66/1246)) compared to laparoscopic (0.5% (9/1680)) and robotic (0.4% (1/224)) cases (*p* < 0.001 for overall comparison).

Superficial surgical site infections occurred in 20.5% of the open cases versus 6.6% of laparoscopic cases and 7.6% of the robotic cases (*p* < 0.001).

Open resection patients had higher rates of 30‐day reoperation and readmission. Reoperation rates were 11.2% for open surgery versus 6.0% for laparoscopic and 9.1% for robotic surgery (*p* < 0.001). Readmission rates were 8.0%, 5.3% and 6.7%, respectively (*p* = 0.023), indicating more postoperative complications with open surgery.

### Anastomotic techniques

Primary anastomosis was performed in 90.3% of cases, while 9.7% underwent stoma formation. Hartmann's procedure accounted for 5.4% (*n* = 186) and abdominoperineal excision (APER) for 5.1% (*n* = 177). Hartmann's procedures were mostly open (70.4%), while APER procedures were mainly minimally invasive (11.9% robotic, 46.9% laparoscopic).

### Anastomotic leakage

**FIGURE 2:**
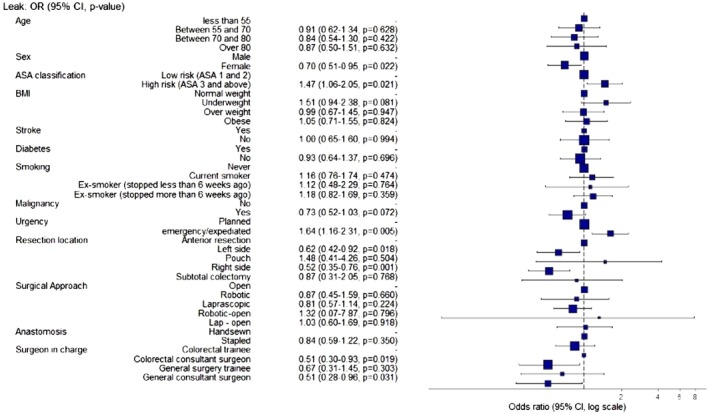
Forest plot of risk factors for anastomotic leakage.

The overall incidence of anastomotic leaks within the study cohort was 7.96%, identified in 214 of 2689 patients who underwent resections with intestinal continuity (Table 3). The leak rate showed significant variability and was associated with specific patient‐ and procedure‐related factors (Table 4, Figure 2). The leak rates for right, left, anterior rectal resection, pouch and subtotal colectomy were 6.9%, 7.7%, 9.7%, 16.0% and 11.8%, respectively (*p* = 0.099). There were no statistically significant differences in the leak rates between handsewn and stapled anastomoses, with rates of 8.8% and 7.7%, respectively (*p* = 0.457).

**TABLE 3:** Patient‐related factors associated with anastomotic leakage following primary anastomosis.

Variable	*N* (total)	levels	No	Yes	Total	*p*
Age	2689 (100.0)	less than 55	548 (90.9)	55 (9.1)	603 (100)	0.657
		Between 55 and 70	932 (92.2)	79 (7.8)	1011 (100)	
		Between 70 and 80	687 (92.7)	54 (7.3)	741 (100)	
		Over 80	308 (92.2)	26 (7.8)	334 (100)	
Sex	2689 (100.0)	Male	1363 (90.7)	139 (9.3)	1502 (100)	0.006
		Female	1112 (93.7)	75 (6.3)	1187 (100)	
ASA class	2689 (100.0)	Low risk (ASA 1 and 2)	1689 (92.9)	129 (7.1)	1818 (100)	0.021
		High risk (ASA 3 and above)	786 (90.2)	85 (9.8)	871 (100)	
BMI	2689 (100.0)	Normal weight	725 (92.8)	56 (7.2)	781 (100)	0.103
		Underweight	259 (88.4)	34 (11.6)	293 (100)	
		Overweight	794 (92.4)	65 (7.6)	859 (100)	
		Obese	697 (92.2)	59 (7.8)	756 (100)	
History of IHD / stroke	2689 (100.0)	Yes	274 (90.7)	28 (9.3)	302 (100)	0.434
		No	2201 (92.2)	186 (7.8)	2387 (100)	
History of diabetes mellitus	2689 (100.0)	Yes	424 (91.2)	41 (8.8)	465 (100)	0.510
		No	2051 (92.2)	173 (7.8)	2224 (100)	
Smoking	2686 (99.9)	Never	1522 (92.9)	117 (7.1)	1639 (100)	0.250
		Current smoker	345 (90.3)	37 (9.7)	382 (100)	
		Ex‐smoker (stopped less than 6 weeks ago)	77 (90.6)	8 (9.4)	85 (100)	
		Ex‐smoker (stopped more than 6 weeks ago)	528 (91.0)	52 (9.0)	580 (100)	
		(Missing)	3 (100.0)	0 (0.0)	3 (100)	
Malignancy	2689 (100.0)	No	562 (89.8)	64 (10.2)	626 (100)	0.021
		Yes	1913 (92.7)	150 (7.3)	2063 (100)	
Urgency	2689 (100.0)	Planned	1986 (92.9)	151 (7.1)	2137 (100)	0.001
		emergency/expediated	489 (88.6)	63 (11.4)	552 (100)	
Resection location	2689 (100.0)	Anterior resection	579 (90.3)	62 (9.7)	641 (100)	0.099
		Left side	717 (92.3)	60 (7.7)	777 (100)	
		Pouch	21 (84.0)	4 (16.0)	25 (100)	
		Right side	1113 (93.1)	82 (6.9)	1195 (100)	
		Subtotal colectomy	45 (88.2)	6 (11.8)	51 (100)	
Surgeon in charge	2689 (100.0)	Colorectal trainee	120 (88.2)	16 (11.8)	136 (100)	0.159
		Colorectal consultant surgeon	1616 (92.6)	129 (7.4)	1745 (100)	
		General surgery trainee	131 (89.1)	16 (10.9)	147 (100)	
		General consultant surgeon	608 (92.0)	53 (8.0)	661 (100)	
Approach	2689 (100.0)	Open	731 (90.5)	77 (9.5)	808 (100)	0.179
		Robotic	170 (92.4)	14 (7.6)	184 (100)	
		Laparoscopic	1360 (93.2)	100 (6.8)	1460 (100)	
		Robotic‐open	7 (87.5)	1 (12.5)	8 (100)	
		Lap – open	207 (90.4)	22 (9.6)	229 (100)	
Anastomosis	2689 (100.0)	Hand‐sewn	485 (91.2)	47 (8.8)	532 (100)	0.457
		Stapled	1990 (92.3)	167 (7.7)	2157 (100)	

**TABLE 4:** Univariate and multivariate analyses of risk factors for anastomotic leakage.

Dependent: Leak		No	Yes	OR (univariable)	OR (multilevel)
Age	Less than 55	548 (90.9)	55 (9.1)	‐	‐
	Between 55 and 70	932 (92.2)	79 (7.8)	0.84 (0.59–1.22, *p* = 0.358)	0.93 (0.63–1.37, *p* = 0.710)
	Between 70 and 80	687 (92.7)	54 (7.3)	0.78 (0.53–1.16, *p* = 0.222)	0.90 (0.58–1.41, *p* = 0.653)
	Over 80	308 (92.2)	26 (7.8)	0.84 (0.51–1.36, *p* = 0.486)	0.91 (0.51–1.61, *p* = 0.740)
Sex	Male	1363 (90.7)	139 (9.3)	‐	‐
	Female	1112 (93.7)	75 (6.3)	0.66 (0.49–0.88, *p* = 0.005)	0.69 (0.51–0.95, *p* = 0.023)
ASA class	Low risk (ASA 1 and 2)	1689 (92.9)	129 (7.1)	‐	‐
	High risk (ASA 3 and above)	786 (90.2)	85 (9.8)	1.42 (1.06–1.88, *p* = 0.017)	1.27 (0.90–1.80, *p* = 0.166)
BMI	Normal weight	725 (92.8)	56 (7.2)	‐	‐
	Underweight	259 (88.4)	34 (11.6)	1.70 (1.08–2.65, *p* = 0.021)	1.58 (0.98–2.55, *p* = 0.059)
	Overweight	794 (92.4)	65 (7.6)	1.06 (0.73–1.54, *p* = 0.759)	1.01 (0.69–1.48, *p* = 0.958)
	Obese	697 (92.2)	59 (7.8)	1.10 (0.75–1.61, *p* = 0.637)	1.10 (0.74–1.65, *p* = 0.626)
History of IHD / stroke	Yes	274 (90.7)	28 (9.3)	‐	‐
	No	2201 (92.2)	186 (7.8)	0.83 (0.55–1.28, *p* = 0.371)	0.93 (0.59–1.47, *p* = 0.761)
History of diabetes mellitus	Yes	424 (91.2)	41 (8.8)	‐	‐
	No	2051 (92.2)	173 (7.8)	0.87 (0.62–1.26, *p* = 0.452)	0.95 (0.65–1.40, *p* = 0.803)
Smoking	Never	1522 (92.9)	117 (7.1)	‐	‐
	Current smoker	345 (90.3)	37 (9.7)	1.40 (0.94–2.04, *p* = 0.092)	1.16 (0.76–1.77, *p* = 0.492)
	Ex‐smoker (stopped less than 6 weeks ago)	77 (90.6)	8 (9.4)	1.35 (0.59–2.71, *p* = 0.432)	1.19 (0.55–2.61, *p* = 0.656)
	Ex‐smoker (stopped more than 6 weeks ago)	528 (91.0)	52 (9.0)	1.28 (0.90–1.79, *p* = 0.155)	1.26 (0.87–1.82, *p* = 0.221)
Malignancy	No	562 (89.8)	64 (10.2)	‐	‐
	Yes	1913 (92.7)	150 (7.3)	0.69 (0.51–0.94, *p* = 0.017)	0.80 (0.56–1.14, *p* = 0.208)
Urgency	Planned	1986 (92.9)	151 (7.1)	‐	‐
	emergency/expediated	489 (88.6)	63 (11.4)	1.69 (1.24–2.30, *p* = 0.001)	1.58 (1.10–2.27, *p* = 0.013)
Resection location	Anterior resection	579 (90.3)	62 (9.7)	‐	‐
	Left side	717 (92.3)	60 (7.7)	0.78 (0.54–1.13, *p* = 0.193)	0.56 (0.37–0.85, *p* = 0.006)
	Pouch	21 (84.0)	4 (16.0)	1.78 (0.51–4.86, *p* = 0.305)	1.39 (0.43–4.48, *p* = 0.577)
	Right side	1113 (93.1)	82 (6.9)	0.69 (0.49–0.97, *p* = 0.034)	0.51 (0.35–0.76, *p* = 0.001)
	Subtotal colectomy	45 (88.2)	6 (11.8)	1.25 (0.46–2.83, *p* = 0.630)	0.87 (0.34–2.24, *p* = 0.768)
Approach	Open	731 (90.5)	77 (9.5)	‐	‐
	Robotic	170 (92.4)	14 (7.6)	0.78 (0.42–1.37, *p* = 0.416)	0.75 (0.39–1.44, *p* = 0.387)
	Laparoscopic	1360 (93.2)	100 (6.8)	0.70 (0.51–0.95, *p* = 0.023)	0.75 (0.52–1.07, *p* = 0.114)
	Robotic‐open	7 (87.5)	1 (12.5)	1.36 (0.07–7.76, *p* = 0.777)	1.18 (0.14–10.07, *p* = 0.883)
	Lap – open	207 (90.4)	22 (9.6)	1.01 (0.60–1.63, *p* = 0.972)	1.02 (0.60–1.73, *p* = 0.940)
Anastomosis	Hand‐sewn	485 (91.2)	47 (8.8)	‐	‐
	Stapled	1990 (92.3)	167 (7.7)	0.87 (0.62–1.23, *p* = 0.405)	0.86 (0.58–1.26, *p* = 0.428)
Surgeon in charge	Colorectal trainee	120 (88.2)	16 (11.8)	‐	‐
	Colorectal consultant surgeon	1616 (92.6)	129 (7.4)	0.60 (0.35–1.08, *p* = 0.068)	0.50 (0.28–0.90, *p* = 0.021)
	General surgery trainee	131 (89.1)	16 (10.9)	0.92 (0.44–1.92, *p* = 0.815)	0.58 (0.26–1.30, *p* = 0.189)
	General consultant surgeon	608 (92.0)	53 (8.0)	0.65 (0.37–1.22, *p* = 0.160)	0.52 (0.27–1.01, *p* = 0.053)

*Note*: Number in model = 2686, Number of groups = 52, AIC = 1476.4, C‐statistic = 0.708.

In the multivariable analysis, female sex (6.3% vs. 9.3%, OR = 0.69, 95% CI 0.51–0.95, *p* = 0.023) and consultant colorectal surgeon (colorectal trainee vs. colorectal consultant vs. general surgery trainee vs. general surgery consultant = 11.8%, 7.4%, 10.9%, 8.0%, OR = 0.50, 95% CI 0.28–0.90, *p* = 0.021 for colorectal consultant vs. colorectal trainee) were associated with a lower leakage rate, whereas emergency cases were associated with a higher leakage rate (11.4% vs. 7.1%, OR = 1.58, 95% CI 1.10–2.27, *p* = 0.013). The leak rate was significantly lower for right‐sided (odds ratio [OR] = 0.51, 95% CI 0.35–0.76, *p* = 0.001) and left‐sided (OR = 0.56, 95% CI 0.37–0.85, *p* = 0.006) resections than for anterior rectal resections.

## DISCUSSION

This large international audit provides a contemporary snapshot of colorectal resection outcomes and allows comparisons with previous ESCP audits. Emerging themes include the widespread adoption of minimally invasive surgery, a plateau in anastomotic leak rates despite technical advances and the critical role of specialised training and quality‐improvement programmes in driving future improvements in patient outcomes.

The overall 30‐day mortality rate of 2.38% is reassuring and indicative of improvements in surgical and perioperative care. Compared to previous ESCP audits, such as the 2015 right colectomy audit, which reported a 30‐day mortality rate of 2.6% [8], our findings suggest a sustained low mortality rate despite the inclusion of a broader range of colorectal resections and emergency cases.

### Anastomotic leak: Stable incidence and ongoing challenge

Despite these advancements, the 30‐day anastomotic leak rate of 7.96% remains a critical concern. This figure is consistent with the rates reported in other large series [17, 18, 19, 20, 21, 22] and previous ESCP audits [10]. For right‐sided resections, the 2015 ESCP audit revealed a 7.4% leak rate [8]. Despite numerous incremental advances, improved stapling devices, tissue sealants and perfusion assessment tools, such as indocyanine green fluorescence, the incidence of leaks in large practices remains stable at approximately 1 in 12 patients with anastomosis. This resonates with prior observations that AL rates have not substantially improved over the past decades [23].

The persistently high incidence of AL underscores the need for continued research and targeted interventions. The factors contributing to AL are multifactorial, encompassing patient‐related factors (e.g. comorbidities and nutritional status), disease‐related factors (e.g. inflammatory conditions and urgency of surgery) and technical factors (e.g. surgical technique, anastomotic tension, blood supply and stapler failure). Its association with emergency surgery is well established, possibly due to factors such as patient instability, suboptimal bowel preparation and the urgency of the procedure precluding patient optimisation. Our analysis reiterates the known risk factors: male sex, likely due to a thicker mesentery or narrower pelvis; low pelvic anastomoses, which have inherently poorer perfusion and are under tension; emergency surgery; and conversion, which may reflect a more challenging procedure or intraoperative complications. These factors were also identified in a 2017 snapshot analysis of left‐sided stapled anastomoses [13].

Notably, age and comorbidity (ASA) were not independent predictors of leakage, although they can affect overall complications. Importantly, anastomotic leak rates did not differ between the open and minimally invasive surgery (MIS) approaches in the audit, indicating that laparoscopy and robotics do not improve anastomotic healing. This finding aligns with the literature, where numerous studies have reported no significant difference in the incidence of leaks between laparoscopic and open colorectal resections [24]. For instance, a meta‐analysis of older adult patients with colon cancer reported equivalent leak rates for laparoscopic versus open resection (OR = 0.91, *p* = 0.71) [24]. Thus, surgeons can be reassured that the benefits of MIS can be achieved without increasing the risk of leaks.

### Impact of specialisation and minimally invasive techniques

Our data clearly demonstrate that MIS has become the standard of care for most elective colorectal resections in many parts of the world by 2022. 55.2% of the cases in this audit were approached laparoscopically or robotically, which has increased from the previous decade. The data identified in CORREA 2022 align with national studies [25, 26, 27, 28], indicating that MIS rates have increased to over 75% in high‐income countries (HICs) [29]. In low‐ and middle‐income countries, the adoption of robotic surgery is less widespread than in HICs, primarily because of challenges in infrastructure, training and costs [30, 31]. Nevertheless, the contributions from LMICs in this field are significant, particularly in the development of new robotic platforms [30, 31]. The widespread adoption of MIS techniques highlights the need for continued training and skill development to ensure optimal patient access and outcomes.

The benefits of MIS in reducing postoperative pain, ileus, wound complications and length of stay are well established, and our findings reinforce these benefits on an international scale [28, 32, 33, 34, 35, 36]. It is worth noting that while MIS offers clear short‐term advantages, we did not observe an independent protective effect of MIS on anastomotic leak in the adjusted analysis; leak rates were similar, provided that the operation could be completed minimally invasively. This suggests that although MIS can reduce certain complications, it does not inherently change the biology of anastomotic healing. Rather, successful MIS is likely a marker of surgical expertise and favourable conditions; when MIS fails (i.e., conversion), it often signals difficulty that correlates with a higher risk of anastomotic leakage [13, 37]. The significantly lower postoperative morbidity and mortality observed with laparoscopic and robotic surgery in this audit are consistent with extensive evidence that minimally invasive colorectal resection improves short‐term outcomes. Meta‐analyses have demonstrated that laparoscopy, when compared to open surgery, reduces the overall rate of complications [38]. In the current cohort, we observed a disparity in mortality rates, with 5% in open cases compared to less than 1% in the MIS group. This discrepancy may be attributed to the inherent selection of more severe cases for the open approach in real‐world scenarios.

The reduction in SSIs associated with MIS techniques (approximately 7% compared to 20% with open surgery) underscores the well‐documented advantages of MIS. The smaller incisions and reduced tissue exposure inherent to MIS contribute to fewer wound complications. Conversely, the use of open surgical approaches in emergency situations is associated with a higher risk of SSIs, which is attributable to the complexity of these cases. This finding aligns with a systematic review that found that laparoscopic colectomy significantly lowered wound infection rates compared to open surgery [38]. Diminished SSI risk not only improves patient recovery but also suggests potential cost savings and faster return to function with MIS approaches.

The inferior outcomes of the open surgery cohort must be interpreted considering the case mix, with open cases being older, sicker and more often emergent. These factors inherently carry higher complication and mortality risks, independent of the surgical technique used [39]. The current analysis incorporated adjusted outcomes based on the surgical approach solely for anastomotic leakage, without conducting a separate multivariate analysis for mortality or overall complications by approach. The mortality rate is higher in emergency colorectal surgery than that in elective cases. The audit reflects this, as many open cases were urgent. However, it is noteworthy that even in high‐risk settings, adopting laparoscopy when feasible can improve outcomes. A recent meta‐analysis of emergency colorectal procedures found that laparoscopy was associated with significantly lower mortality (OR 0.44, 95% CI 0.35–0.54) and morbidity (OR 0.53, 95% CI 0.43–0.65) than open surgery [38]. This suggests that some of the worse outcomes in open cases might be mitigated by the broader use of minimally invasive approaches in appropriately selected high‐risk or urgent patients. In practice, expanding MIS to frail or emergency patients when anatomically and haemodynamically feasible could confer survival and recovery benefits.

Robotic surgery has shown a gradual uptake, particularly for rectal cancer. Our audit shows that robot‐assisted resections comprised less than 5% of cases globally in 2022, reflecting cost and access issues, as well as a still‐maturing evidence base for this technology. In our audit, the higher utilisation of neoadjuvant therapy among rectal cancer patients treated robotically (27.2% vs. 14.3% laparoscopic and 11.6% open; *p* < 0.001) likely reflects both case selection for more complex, downstaging‐requiring disease and the implementation of robotic capability within centres with mature multidisciplinary tumour boards and streamlined access to neoadjuvant therapy.

In colon surgery, randomised trials are sparse, but large observational series (including Dutch audit data) indicate comparable outcomes between robotic and laparoscopic colectomies, with the main difference being a lower conversion rate in robotic cases (right, left and sigmoid colectomies: 4.6% vs. 8.8%, 4.6% vs. 11.6% and 1.6% vs. 5.9%, respectively) [29].

In rectal cancer, where the technical difficulty is higher, the ROLARR trial found no significant difference in positive margin rates between robotic and laparoscopic total mesorectal excision, although conversions were fewer with robotics (8.1% vs. 12.2%) and no statistically significant differences were found for intraoperative and postoperative complications, surgical plane, 30‐day mortality and urinary and sexual dysfunction [40].

Recent data have been derived from the REAL (Robotic versus Laparoscopic Surgery for Mid–Low Rectal Cancer) randomised controlled trial, which included 1171 patients in an intention‐to‐treat analysis [41]. Compared with laparoscopy, the robotic approach was associated with a lower rate of positive circumferential resection margins (4.0% vs. 7.2%; *p* = 0.023) and fewer abdominoperineal resections (16.9% vs. 22.7%). It also yielded fewer conversions to open surgery (1.7% vs. 3.9%; *p* = 0.021), a lower proportion of patients with Clavien–Dindo grade ≥ II complications (16.2% vs. 23.1%; *p* = 0.003) and a shorter length of hospital stay (7.0 vs. 8.0 days; *p* = 0.0001) [41].

Our data confirmed a significantly lower conversion rate for the robotic approach than for laparoscopy (4.3% vs. 14.6%). As robotic platforms become more widely available (and competing systems potentially reduce costs), the proportion of robotic colorectal procedures is likely to increase.

In summary, the adoption of minimally invasive surgery (MIS) has improved recovery and lowered rates of superficial complications following colorectal resection. Within MIS, robotic surgery offers further benefits, including a shorter length of stay and fewer conversions compared with laparoscopy. However, the key challenge of anastomotic integrity transcends the choice of MIS versus open surgery, hinging on patient factors, surgical judgement and meticulous surgical techniques.

### Quality‐improvement initiatives

The persistence of AL as a major problem has generated targeted quality‐improvement programmes, such as the ESCP EAGLE trial [42]. The results of the EAGLE, a cluster‐randomised study conducted in 64 countries, are highly informative. In hospitals that achieved high compliance with EAGLE training (≥80% of surgeons completed the modules), leak rates dropped dramatically from 12.2% to 5.1% (OR 0.36, *p* < 0.001) [42]. Our 2021 audit occurred contemporaneously with the EAGLE intervention period (2020–2022); thus, some high‐performing centres in our data may have already reflected EAGLE‐influenced practices, potentially contributing to the slight improvement in the leak rate among the engaged surgeons. Perioperative care optimisation is another aspect of surgical quality improvement. Interestingly, a sub‐analysis of the 2017 audit showed that patients who received mechanical plus oral antibiotic prep had lower leak rates (approximately 6.1%) than those who received mechanical prep alone or no prep [8]. The persistently high rates of anastomotic leaks underscore the urgent need for intensified research into predictive biomarkers, novel preventive strategies and improved management protocols for AL. Collaborative international efforts, such as those facilitated by the ESCP, are essential for driving quality‐improvement initiatives and disseminating best practices globally. The identified risk factors for AL can aid clinicians in preoperative risk stratification and patient counselling, allowing for more personalised surgical planning and enhanced vigilance in high‐risk individuals. Policymakers should consider these benchmarks when developing national and international guidelines to improve colorectal surgical care.

Differences in surgeon specialisation may have contributed to the observed outcome patterns [13]. In this audit, most MIS cases were managed by colorectal specialist surgeons, whereas many open surgeries were performed by general surgeons. Higher surgeon expertise and volume are well correlated with better colorectal outcomes. For example, patients operated on by board‐certified colorectal surgeons have significantly better survival rates than those treated by non‐specialists [43]. Specialty‐trained surgeons also achieve more thorough oncological resections (e.g., higher lymph node yields) [43]. The superior outcomes observed in the MIS group may partly reflect the involvement of specialists.

**Implications for practice**

The CORREA 2022 audit has implications for future studies. First, the high use of minimally invasive techniques and comparable oncologic outcomes should encourage the continued expansion of MIS training. Second, the consistently high rate of anastomotic leaks demonstrates a need for emphasis on decision‐making processes and perioperative optimisation concerning patient, disease and medical factors rather than minimally invasive approach alone. This means adhering to the principles of good blood supply (no tension, wide mobilisation and preserving arcades), testing the anastomosis and considering a protective stoma for high‐risk distal anastomoses. Third, the influence of conversion on worse outcomes suggests that patient selection and surgeon experience remain crucial. Conversion in difficult cases is sometimes unavoidable and is indeed the correct decision for patient safety [37]. However, careful preoperative planning, early involvement of senior surgeons or choosing an open approach upfront in hostile abdomens may mitigate some conversions. Moreover, by reducing conversion in some cases, the robotic platform could indirectly improve outcomes if targeted to cases where laparoscopy is marginal [44, 45, 46].

### Limitations

It is important to acknowledge that this audit, although comprehensive, has some inherent limitations. Participation was voluntary and thus may have been biased towards centres with an interest in outcomes (potentially higher‐performing centres). We attempted to achieve broad participation, including many mid‐volume regional hospitals; however, some countries and hospitals may have been underrepresented. Furthermore, while the audit provides valuable insights into short‐term outcomes, it does not capture long‐term outcomes, such as recurrence rates for malignancy or long‐term quality of life, which are crucial for a complete understanding of surgical effectiveness. Future audits should aim to collect long‐term outcome data, including oncological outcomes and patient‐reported quality of life, to provide a more comprehensive understanding of the impact of colorectal surgery. Nonetheless, the strength of an audit is its real‐world, international and prospectively collected data; thus, the design enhances the generalisability of the findings.

## CONCLUSIONS

The CORREA 2022 ESCP audit revealed a field in transition: technical approaches in colorectal surgery have advanced markedly, with MIS now predominant across much of the globe; however, fundamental outcome metrics, such as anastomotic leakage, have not significantly changed at the population level. This study confirms the known risk factors and highlights the ongoing need for concerted quality‐improvement efforts.

## AUTHOR CONTRIBUTIONS

**2022 European Society of Coloproctology ESCP CORREA Collaborating Group:** Conceptualization; investigation; funding acquisition; methodology; validation; visualization; writing – review and editing; formal analysis; project administration; data curation; supervision; resources; software.

## FUNDING INFORMATION

This research received no specific grant from any funding agency in the public, commercial, or not‐for‐profit sectors.

## CONFLICT OF INTEREST STATEMENT

The authors have nothing to declare.

## Data Availability

The data that support the findings of this study are available on request from the corresponding author. The data are not publicly available due to privacy or ethical restrictions.

## APPENDIX A

**2022 European Society of Coloproctology (ESCP) CORREA Collaborating Group membership**:

**Writing group**:

Ionut Negoi, Gabrielle van Ramhorst, Shaji Sebastian, Gianluca Pellino, Muhammed Elhadi, Audrius Dulskas, Bryar Kadir, James Glasbey, Peter Neary, Ana María Minaya Bravo, James Keatley, Alaa El‐Hussuna*, Thomas Pinkney*, Sanjay Chaudhry* (lead for CORREA audit).

*joint senior authors.

**Data collation, oversight and analysis (University of Birmingham):**

Bryar Kadir, James Keatley, Laura Magill, Rita Perry.

**Cohort Studies Working Group**:

Alaa El‐Hussuna (chair), Sue Blackwell, Sanjay Chaudhri, Sharfuddin Chowdhury, Dragomir Dardanov, Audrius Dulskas, Muhammed Elhadi, Caterina Foppa, Gaetano Gallo, James Glasbey, Elizabeth Li, Ana María Minaya Bravo, Dion Morton, Peter Neary, Ionut Negoi, Francesco Pata, Gianluca Pellino, Thomas Pinkney, Gabrielle van Ramshorst, Shaji Sebastian, Beatriz Silva Mendes, Baljit Singh.

**ESCP research committee:**

Thomas Pinkney (chair), Erman Aytac, Sue Blackwell, Stephanie Breukink, Pamela Buchwald, Niki Christou, Dragomir Dardanov, Alaa El‐Hussuna, Caterina Foppa, Gaetano Gallo, Nir Horesh, Karoline Horisberger, James Keatley, Jim Khan, Laura Koskenvuo, Yurij Košir, Hans Lederhuber, Dion Morton, Gabrielle van Ramshorst, Mostafa Shalaby, Carolynne Vaizey.

**Site‐level Collaborators:**

Albania: S. Bilali, S. Ferko (Durres Regional Hospital); A. Gjata, E. Shehi, J. Shahu (University Hospital Center Nene Tereza).

Algeria: S. Bouaoud, M. Abdoun, K. Bouchenak, H. Saada (CHU Saadna Abdennour – Emergency); A. Ouyahia, M. Rais, E. Seddik, M. Bouaoud, A. Kouicem (CHU Saadna Abdennour – General); N. Yacine, L. Nawel, M. Abdoun, N. Talbi (Centre hospitalo‐universitaire spécialisé de lutte contre le cancer); A. Tidjane, B. Tabeti, A. Hebbar (EHU‐1st November 1954); I. Lalmi, A. Bouanani, O. Bali, N. Benallel (Public hospital establishment of Sig).

Argentina: C. Chwat, G. Lemme, G. Rosato, F. Alexandre, D. Valli (Hospital Universitario Austral).

Aruba: M. Gosselink, D. Daryanani, J. Smit, A. Ponson, Y. Casseres (Dr. Horacio E. Oduber Hospital).

Australia: N. Strugnell, B. D'Souza, S. Badiani, T. Kuany (Northern Hospital).

Austria: F. Aigner, M. Mitteregger, S. Uranitsch, C. Allmer, G. Moitzi, E. Wallner, R. Stadler (Barmherzige Brüder Graz); F. Herbst, I. Iliev (St. John of God Hospital, Vienna).

Azerbaijan: E. Samadov, A. Ibrahimli (Leyla Medical Center).

Bahrain: M. Saeed, R. Varkey, B. Ebrahim, H. Aljazaf, I. Juma (King Hamad University Hospital).

Belgium: B. Monami, S. Markiewicz, D. Francart, F. Jehaes, N. Debergh (CHC MontLegia Liège); P. Malvaux, S. Grandjean (Hospital Center of Wallonie Picarde); J. Stijns, E. Van Eetvelde, T. Van de Winkel, S. Violon (UZ Brussel); G. van Ramshorst, S. Tavares Sousa Castro, D. Van de Putte, Y. Van Nieuwenhove, P. Pattyn (University Hospital of Ghent).

Bulgaria: T. Ivanov, E. Filipov, D. Georgiev, V. Neykov (Heart and Brain Hospital Pleven); D. Dardanov, M. Sokolov, S. Maslyankov, S. Toshev, P. Gribnev, A. Koychev (University Hospital Alexandrovska); M. Karamanliev, D. Dimitrov, P. Vladova, M. Shoshkova, A. Shanker (University Hospital Dr. Georgi Stranski); M. Slavchev, N. Belev, R. Penkov, B. Atanasov, P. Krastev (University Hospital Eurohospital); I. Yotsov (University Hospital Medika).

Croatia: G. Šantak (General County Hospital Požega).

Cyprus: N. Gouvas, P. Papatheodorou, K. Konstantinou, G. Kokkinos, A. Stylianou (Nicosia General Hospital).

Denmark: P. Christensen, R. Balachandran, J. Eriksen, M. Kristensen, S. Ingvarsdóttir (Aarhus University Hospital); M. Ellebaek, J. Baelum, M. Kjær, P. Cuk, I. Al‐Najami (Odense and Svendborg University Hospital); U. Løve, P. Fink, T. Eliasen, D. Zavrakidis, S. Lynge, L. Sjælland (Viborg Regional Hospital).

Egypt: M. Abdel‐Maboud, S. Mahmoud, H. Abozied (Al Hussein University Hospital); M. Al Sayed, A. Swealem, A. Elshinnawy, S. Mahmoud, A. Amer, M. Ewedah (Alexandria Main University Hospital); M. Shalaby, H. Elfeki, A. Sakr, A. Sanad, M. Sadek (Mansoura University Hospital); K. Abdelwahab, I. Nazif, H. Aboelfarh (Mansoura health insurance hospital); B. Refky, M. Shetiwy, I. Metwally, M. Abdelkhalek, M. Zuhdy (Oncology Center Mansoura University); M. Omar, A. Safy, M. Alansary (Qena University Hospital).

France: B. Trilling, J. Faucheron, F. Tidadini, L. Audeguy, Q. Chenevas‐Paule, A. Roudier (CHU Grenoble‐Alpes); N. de Angelis, C. Schena, G. Bianchi (Henri Mondor University Center); N. Christou (Limoges Hospital).

Germany: A. Fürst, M. Brunner, S. Blümel, V. Schmidt (Caritas Krankenhaus St. Josef Regensburg); A. Krappitz, A. Türler, M. Derenbach, W. Lydia (Johanniter Krankenhaus Bonn); J. Baral, S. Muench, F. Pullig, R. Langbecker (Klinikum Karlsruhe); D. Jauch, S. Fichtner‐Feigl, H. Neeff, C. Berlin, L. Matuschik (University Hospital Freiburg); J. Kleeff, U. Ronellenfitsch, O. Bayram, J. Klose, S. Rieder (University Hospital Halle (Saale)).

Greece: D. Korkolis, A. Sarafi, E. Fradelos, G. Kavalieratos, I. Katsoulis (Agios Savvas Anticancer Hospital); I. Papaconstantinou, M. Konstadoulakis, T. Theodosopoulos, A. Gklavas, D. Politis (Aretaieion Hospital); T. Petropoulou, A. Polidorou (Athens Euroclinic); A. Ioannidis, M. Konstantinidis, S. Konstantinidou, K. Konstantinidis (Athens Medical Center); D. Manatakis, C. Barkolias, D. Balalis, N. Tasis (Athens Naval and Veterans Hospital); S. Kapiris, A. Kolinioti, M. Sotiropoulou, M. Psarologos, P. Metaxas (Evaggelismos General Hospital); A. Papadopoulos, E. Manioti, O. Mouzakis, D. Broutas, V. Nikolaou (General Hospital of Nikaia); G. Tzovaras, I. Baloyiannis, K. Perivoliotis, I. Mamaloudis, F. Bobou (General University Hospital of Larissa); K. Bouchagier, G. Skroubis, I. Maroulis, F. Mulita, E. Abalov (General University Hospital of Patras); O. Ioannidis, L. Loutzidou, E. Anestiadou, S. Bitsianis, S. Symeonidis (George Papanikolaou General Hospital of Thessaloniki); G. Theodoropoulos, M. Frountzas (Hippocration General Hospital – First Propaedeutic); S. Volteas, N. Dimitriou, D. Thermou, G. Karantzikos, S. Kympouris (Hippocration General Hospital – Site B); C. Mavrantonis, P. Alexakou, A. Karagouni, N. Boltsis, D. Paradellis (Hygeia hospital); D. Schizas, I. Karavokyros, N. Machairas, A. Syllaios, S. Kykalos (Laiko University Hospital); K. Stamou, G. Tsiotos, D. Fanidou (Mitera Hospital); E. Kontis, I. Katsaros, E. Kaouras, L. Katsiaras, P. Manikis (Metaxa Cancer Hospital of Piraeus); N. Zampitis, A. Marinis, E. Bourmpouteli, M. Merrakos, F. Stefou (Tzaneio General Hospital).

Hungary: B. Banky, P. Ónody, L. Lakatos, Á. Dániel, V. Bencze, A. Fülöp (1st Department of Surgery– Semmelweis University).

Iceland: E. Valsdottir, J. Atladottir, H. Sigurdsson, H. Einarsdottir, P. Moller (Landspitali National Hospital).

India: A. Saklani, M. Kazi (Tata Memorial Hospital).

Ireland: D. Kearney, D. Petrocz‐Turu, C. O'Reily (Connolly Hospital Blanchardstown); S. Martin, R. Kennelly, F. Lecot, I. Reynolds (St Vincent's Private Hospital); D. Winter, A. Hanly, S. Arya, D. Hechtl (St Vincent's University Hospital); J. O'Riordan, É. Ryan, J. Mahon, D. Kavanagh, N. Christodoulou (Tallaght Hospital); P. Neary, B. Creavin, F. Cooke, P. McCullough, H. Earley (University Hospital Waterford/University College Cork).

Israel: I. Kent, N. Horesh, A. Mansur, M. Kyzer, R. Antebi (Sheba Medical Center).

Italy: R. Tutino, M. Santarelli, D. Visconti, A. Ferguglia, L. Rorato (AOU Città della Salute e della Scienza); M. Annecchiarico, G. Argenio (Azienda Ospedaliera San Pio); C. Pedrazzani, G. Turri, C. Conti, G. Gecchele, E. De Giulio, N. Bicelli (Azienda Ospedaliera Universitaria Integrata di Verona); R. Balestri, M. Puccini, P. Buccianti, R. D'Ischia, D. Pezzati (Azienda Ospedaliero Universitaria Pisana); L. Marano, L. Carbone, L. Verre, F. Roviello, R. Piagnerelli (Azienda Ospedaliero Universitaria Senese); D. Scala, B. Puppio, P. Maione, G. Marino (Basilicata Cancer Referral Center); B. Scotto, F. Ascari, S. Muratori, I. Roli (Bernardino Ramazzini Hospital); V. Tonini, M. Cervellera, J. Shahu, L. Sartarelli (Central Hospital of Taranto – SS. Annunziata); A. Lauretta, S. Pollesel, M. Olivieri, C. Belluco (Centro di Riferimento Oncologico di Aviano (CRO) IRCCS); M. Calabrò, A. Muratore, B. Cuzzola, E. Herranz Van Nood, M. De Zuanni (Edoardo Agnelli); M. Manigrasso, G. De Palma, M. Milone, S. Vertaldi, A. Marello (Federico II University of Naples); L. Solaini (Morgagni‐Pierantoni); A. Luzzi, E. Romairone, S. Carrabetta, P. Grondona, A. Filippelli (Ospedale Villa Scassi); G. Moretto, I. Harmony (Ospedale Pederzoli); F. Bianco, S. Gili, A. Novi, S. Grassia, A. Cappiello (Ospedale S. Leonardo – ASL Napoli 3 sud, Castellammare di Stabia); N. Tamini, L. Cigagna (Ospedale San Gerardo); F. Ghignone, G. Ugolini, L. Santandrea, S. Bolzon (Ospedale Santa Maria delle Croci); C. Marafante, E. Moggia (Ospedale degli Infermi di Rivoli); M. Grande, M. Campanelli, L. Siragusa, F. Santori, G. Sica (Policlinico Tor Vergata Hospital, Rome); U. Grossi, R. Tutino (Regional Hospital ULSS 2 Marca Trevigiana); R. Galleano, A. Langone, M. Ciciliot, M. Malerba (San Paolo); P. De Nardi, A. Vignali, G. Carbone, R. Rosati (San Raffaele Scientific Institute, Milan); M. Rottoli, A. Belvedere, A. Gori, C. Larotonda, S. Cardelli, T. Violante (Sant'Orsola‐Malpighi University Hospital); D. Sasia, M. Giuffrida, E. Beltrami, G. Preve, S. Armentano (Santa Croce e Carle Hospital, Cuneo); P. Mosangini (Santa Maria della Misericordia); M. Ammendola, G. Currò, G. Ammerata, R. Filippo (University ‘Magna Graecia’ of Catanzaro); A. Picciariello, M. De Fazio, L. Vincenti, G. Tomasicchio (University of Bari ‘Aldo Moro’); F. Selvaggi, G. Pellino, M. D'Ambrosio, F. Menegon Tasselli, D. Massaro (Università della Campania ‘Luigi Vanvitelli’, Naples); G. Terrosu, G. Calini, L. Bonello, F. Leone (Santa Maria Della Misericordia).

Jordan: A. Khamees, S. Al‐issawi, S. Abu Wardeh, A. Mansour, A. Mohammad (Al‐Basheer Hospital); O. Shattarah, R. Ebbini, O. Sarhan, M. Nour, F. Daoud (King Hussein Cancer Center).

Kuwait: A. Tarboush (Mubarak Al‐Kabeer Hospital).

Latvia: A. Pcolkins (Riga East CUH Latvia Oncology Center).

Libya: G. Alkadeeki, F. AlMaadany (Al‐Jalaa Hospital); A. Kredan, A. Mohamed, A. Haddud (Aldiaa Hospital); W. Abdulnabi, F. Fauzi, M. Ali, D. Zreeg (Alkhadra Hospital); W. Aldressi, E. Hosain, H. Alkeelani, I. Abuzeid (Benghazi Medical Center); A. Alkaseek, H. Shames, H. Baliad Bakeer (Gharyan Central Hospital); F. Elhajdawe, E. Abdulwahed, E. Alshareea, K. Derwish, E. Soula (Tripoli Central Hospital); M. Zagmen, S. Zagmen, A. Fteis, H. Mansour, H. Gholka (Tripoli Medical Center).

Lithuania: A. Dulskas, J. Kuliavas, Z. Gricius, A. Aleinikov, R. Bradunaite (National Cancer Institute).

Republic of Macedonia: A. Nikolovski, I. Fildishevski, E. Minova (University Surgery Clinic “St. Naum Ohridski”).

Malaysia: M. Mohamad Salmi, A. Md Nor, F. Elagili, M. Sainal, A. Zainudin (International Islamic University Malaysia Medical Centre); F. Hayati, R. Sriram, S. Subramaniam, J. Mah, S. Tan (Queen Elizabeth Hospital, Kota Kinabalu); A. Zakaria, Z. Zakaria, M. Wong, K. Hamdan, S. Dass (School of Medical Sciences and Hospital USM, Universiti Sains Malaysia).

Malta: P. Andrejevic, J. Psaila, C. Cini, J. Schembri Higgins, M. Portelli (Mater Dei Hospital).

Morocco: A. Majbar, A. Souadka, A. Benkabbou, S. Echiguer, M. El Hassouni (Institut National d'Oncologie).

Netherlands: J. Konsten, F. Aarts, F. van Osch, T. Schok (VieCuri Medisch Centrum); E. Boerma, M. Martens, I. Poodt, K. van Dam (Zuyderland Medical Center).

New Zealand: I. Bissett, C. Varghese, W. Xu, G. O'Brien, J. Penfold, M. Alvarez, R. He (Auckland City Hospital); A. Lin, P. Fagan, S. Farrant, S. Jury, J. Canton (Wellington Regional Hospital); C. Harmston, M. McGuinness, W. Xu, H. Witcomb‐Cahill, H. Boyes (Whangarei Hospital).

Nigeria: A. Adeyeye, E. Afekahi (Afe Babalola University Multi‐System Hospital).

Pakistan: T. Chawla, U. Waqar, A. Fatimi, M. Arshad, A. Rehman (Aga Khan University); R. Aziz, S. Haider, A. Hai, S. Ahmed, A. Rehman (Dr. Akbar Niazi Teaching Hospital).

Poland: M. Stanczak (Maritime Hospital).

Portugal: M. Cunha, B. Mendes, I. Miguel, J. Rachadell, E. Amorim (Centro Hospitalar Universitário do Algarve – Unidade de Portimão); C. Carneiro, R. Rocha, F. Almeida, R. Pera, J. Frazão (Hospital Prof. Doutor Fernando Fonseca, E.P.E.); J. Costa Pereira, C. Costa Pereira, O. Oliveira, N. Gonçalves, E. Gonçalves (Hospital de Braga); S. Fortuna Martins, H. Devesa, O. Teslyak, R. Barradas, S. Marisa Marques (Hospital de Santarém); A. Faustino, M. Almeida, D. Acosta, R. Quintanilha (Hospital do Divino Espírito Santo); P. Silva‐Vaz, H. Perez, R. Rainho, R. Monteiro, T. Neves (Unidade Local de Saúde de Castelo Branco).

Qatar: M. Yousif, A. Ahmed, A. Parvaiz, M. Abunada, A. Aleter (Hamad General Hospital).

Romania: M. Dimofte, S. Morarasu, A. Musina, N. Velenciuc, S. Lunca (Regional Institute of Oncology Iasi).

Russian Federation: A. Yanishev, M. Bagrjancev, P. Zarubenko, A. Abelevich, A. Kokobelyan (Privolzhsky Research Medical University, Nizhny Novgorod regional clinical hospital, Sechenov Medical State University); A. Karachun, A. Olkina, T. Lankov, D. Samsonov, A. Petrov (N.N. Petrov National Medical Research Center of Oncology).

Saudi Arabia: S. Chowdhury, S. Alshahrani (King Saud Medical City).

Slovenia: J. Grosek, J. Košir, T. Košir Božič, A. Tomažič (University Medical Centre).

Spain: J. Arredondo, J. Baixauli, C. Pastor, C. Sanchez, A. Alvarellos (Clinica Universidad de Navarra); V. Maderuelo Garcia, A. Huidobro Piriz, C. Suero Rodriguez, E. Castrillo Arconada, H. Ordas Macias (Complejo Asistencial Universitario de Palencia); C. Martinez Perez, M. García Coret, C. Baez Burgos, C. Cifre Martinez, M. Tome Jimenez (Consorcio Hospital General Universitario); R. García‐Domínguez, J. Cutillas‐Abellán, A. Fluixà‐Pelegrí, N. Ridaura‐Capellino, J. Seguí‐Gregori (Francesc de Borja Hospital); S. Serra Pla, T. Labró, C. Soto, C. Gómez, P. Collera (Fundació Althaia – Xarxa Assistencial Universitària de Manresa); J. Ocaña, J. Die, J. Fernandez‐Cebrian, P. Pastor, A. Garcia‐Chiloeches, A. Ballestero, L. Juez (Hospital Universitario Ramon Y Cajal); J. Dziakova, J. Muguerza, D. Rivera Alonso, V. Catalan, M. Sajonia‐Coburgo (Hospital Clinico San Carlos); D. Moro‐Valdezate, V. Pla‐Marti, J. Martin‐Arevalo, A. Espi‐Macias, L. Perez‐Santiago (Hospital Clínico Universitario de Valencia); M. Carrasco, P. Parra, J. Muñoz, E. Peña, M. Ramírez (Hospital General Reina Sofía); P. Tejedor, L. Jimenez (Hospital General Universitario Gregorio Marañón); M. Labalde, P. Pelaez Torres, C. Nevado García, E. Ferrero Herrero, F. Garcia Borda (Hospital Universitario 12 de Octubre); J. Bernal‐Sprekelsen, S. Gómez‐Abril, T. Torres, E. Martí (Hospital Universitario Doctor Peset); N. Borda Arrizabalaga, G. Elorza‐Echaniz, A. Andres‐Imaz, I. Aguirre‐Allende (Hospital Universitario Donostia); M. Duque‐Mallen, C. Gracia‐Roche, M. Sanchez‐Fuentes, A. Martinez‐German, M. Santero‐Ramirez (Hospital Universitario Miguel Servet); M. Diez‐Alonso (Hospital Universitario Principe de Asturias); M. Estaire Gómez, J. Valverde Mantecón, J. Martín Ramiro, E. Cagigal Ortega, M. Cancelas Felgueras (Hospital Universitario Severo Ochoa); E. Colás‐Ruiz, J. Lliteras‐Jorge, M. Tasende‐Presedo, S. Baena‐Bradaschia, L. Lozano‐Salva (Hospital Universitario Son Llàtzer); J. Gomez‐Rosado, J. Valdes‐Hernandez, F. DelRio‐Lafuente, J. Cintas‐Catena, A. Perez‐Sanchez (Hospital Universitario Virgen Macarena); J. Abrisqueta, N. Ibáñez (Hospital Universitario Virgen de la Arrixaca); B. Arencibia‐Pérez, C. Roque‐Castellano, E. Nogués‐Ramia, Y. Sosa‐Quesada, M. Artiles‐Armas (Hospital Universitario de Gran Canaria Doctor Negrín); S. Gortázar de las Casas, I. Pascual Migueláñez, M. Álvarez Gallego, A. Gegundez Simón, B. Monje Vera (Hospital Universitario la Paz); A. Minaya‐Bravo, A. Sanchez‐Gollarte, A. Galvan‐Perez, E. Gonzalez‐Gonzalez (Hospital del Henares); S. Alonso, M. Jiménez, S. Salvans, M. Pascual, B. Montcusí (Hospital del Mar); E. Viejo Martínez, J. Ripolles, M. de Fuenmayor Valera, C. Ortiz Johansson, E. Alvaro Cifuentes (Infanta Leonor University Hospital); E. Gutiérrez Cafranga, R. Escalera Pérez, W. Sánchez Bautista, J. Esteban Ramos, F. García Molina (Jerez de la Frontera University Hospital); L. Cristobal, M. Gomez, C. Cagigas, N. Suarez (Marqués de Valdecilla University Hospital); G. Valero‐Navarro, E. Pellicer‐Franco, V. Soria‐Aledo, M. Mengual‐Ballester, J. Garcia‐Marin (Morales Meseguer University Hospital); A. Solís Peña, C. Petrola Chacón, M. Kraft Carré, F. Marinello, E. Espín Basany (Vall d'Hebron University Hospital); B. De Andrés‐Asenjo, A. Romero, A. Vázquez, G. Cabezudo, J. Beltrán de Heredia (Valladolid University Clinical Hospital).

Sri Lanka: D. Wickramasinghe (National Hospital of Sri Lanka).

Sudan: S. Galal‐Eldin, H. Hamid (Al‐Moalem Medical City); N. Hashim (Khartoum North Teaching Hospital (Bahri)); M. Abdulmajeed, R. Elhadi, E. Abuobaida Bannaga, R. Hashim, F. Osman (Ribat university hospital); H. Fadlalmola, A. Eltayeb, A. Ameen (Soba University Hospital).

Sweden: S. Al Assaad, A. Papp, M. Pelczar (Hudiksvall Hospital); P. Buchwald, M. Lydrup, N. Azhar, T. Vedin, H. Jutesten (Skåne University Hospital Malmö).

Switzerland: F. Ris, E. Liot, N. Buchs, L. Gialamas, A. Litchinko (Geneva University Hospitals); B. Schiltz, C. Viehl, A. Müller, A. Lehnen, C. Regula (Hospital Center Biel); I. Fournier, E. Kalogiannis, S. Gussago, D. Chappalley, B. Guendil (Hôpital de Sion – Centre Hospitalier du Valais Romand (CHVR)); R. Galli, R. Rosenberg, K. Roosen (Kantonsspital Baselland); M. Adamina, G. Peros, P. Müller, M. Giardini, T. Müller (Kantonsspital Winterthur); M. von Strauss und Torney, M. Bolli, A. Polutak, S. Wullschleger, K. Herzog (St Clara Hospital); S. Däster, S. Soysal, F. Haak, G. Hess, B. Wiesler (University Hospital Basel).

Syria: H. Al Houri, A. Alhouri, S. Jomaa, M. Daher, S. Abbas (Al Assad University Hospital).

Syria: M. Klib, A. Hammadieh, M. Ghandour, M. Chikh Salem, O. Danawer (Al‐Mouwasat University Hospital).

Tunisia: M. Chaouch, K. Zouari, T. Kellil (University Hospital Fatouma Bourguiba).

Turkey: E. Aytaç, C. Saracoglu, V. Özben, M. Gulmez, A. Mutlu (Acibadem Atakent Hospital); T. Olmez, A. Seker, A. Sozutek, K. Kaplan (Adana City Training and Research Hospital); S. Zenger, B. Gurbuz, U. Can, T. Yalti, D. Bugra (American Hospital); I. Gecim, M. Koc, C. Akyol, S. Ersoz, A. Erkek (Ankara University Medical School); Y. Altinel, S. Meric, K. Ozdogan, Y. Aktimur, A. Barcin (Bagcilar Research and Training Hospital); E. Balik, E. Ozoran, I. Ozata, D. Bugra, D. Uymaz (Koç University Medical School); C. Ulusoy, A. Demirel, S. Güçlü Mete, M. Atci (Okmeydanı Training and Research Hospital); I. Cakcak, E. Aydoğdu, A. Goztepe, M. Ajredini (Trakya University Hospital); O. Ozkan, H. Ulgur, O. Duzgun, M. Kalin, E. Kirkan (University of Health Sciences Istanbul Umraniye Training and Research Hospital).

Ukraine: A. Kebkalo, M. Boruta, V. Tyselskyi, Y. Tryliskyy, I. Kluzko (Kyiv Regional Clinical Hospital).

United Kingdom: J. On, R. Shearer, M. Elhusseini, S. Coull, F. Carnegie (Aberdeen Royal Infirmary); H. Joshi, J. Davies, C. Simillis, G. Theodoroleas (Addenbrooke's Hospital); T. Hammond, J. Fairbanks, Z. Elliot, T. Khan (Broomfield Hospital); P. Nastro, L. Poynter, S. Nikolaou, R. Bhardwaj, J. Adamek (Darent Valley Hospital); B. Stubbs, G. Neelankavil Davis, J. Eid, Z. Naumowicz, M. Gupta (Dorset County Hospital); J. Sebastian, S. Mangam, J. Evans, G. Preziosi, R. Koshy (East Kent Hospitals NHS Foundation Trust); R. Harshen, M. Badawi, A. Rahman, I. Rakhimov, N. Cruikshank (East Sussex Healthcare (Conquest hospital and Eastbourne District General Hospital)); J. Kynaston, M. Yule, F. Reilly (Forth Valley Royal Hospital); K. Marimuthu, A. John, S. Bhanderi, R. Woods, L. Taylor (George Eliot Hospital); M. Dhruva Rao, M. Afridi, I. Elnagar (Glangwili General Hospital); S. Arnold, F. Di Fabio, A. Venkatasubramaniam, C. Yao, E. Anand (Hampshire Hospitals NHS Trust); A. Myers, Y. Mohsen, A. Prabhudesai, A. Slesser, S. Mallappa (Hillingdon Hospital); G. Bond‐Smith, T. Fung, S. Kok (John Radcliffe Hospital); F. Runau, B. Singh, S. Khan, A. Eltweri, J. Wolff (Leicester General Hospital); K. Boyle, P. Sarmah, I. Kais, H. Merriman (Leicester Royal Infirmary); S. Gurjar, M. Al‐Ani, S. Ahmed, N. Ul‐Ain, H. Tabasi (Luton and Dunstable University Hospital); B. Keeler, F. Dixon, M. Rana (Milton Keynes University Hospital); J. Khan, R. Harvitkar, S. Stefan, R. Duhoky (Queen Alexandra Hospital); R. Bethune, H. Lederhuber, T. Cheung, E. Matthews (Royal Devon and Exeter Hospital); K. Altaf, S. Ahmed, N. Pang, C. Simms, G. Titley‐Wilson (Royal Liverpool University Hospital); M. Thaha, A. Minicozzi, H. Patel, A. Taha (Royal London Hospital); G. Omar, M. Riad (Tunbridge Wells Hospital); C. Magee, S. Small, L. Convie, J. Ong, C. Rossborough (Ulster Hospital Dundonald); N. Chandratreya, J. Noronha, M. Alshibshoubi, E. Dean, J. Thompson (Weston General Hospital).
